# Palladium-Catalyzed Dehydrogenative Coupling: An Efficient Synthetic Strategy for the Construction of the Quinoline Core

**DOI:** 10.3390/md15090276

**Published:** 2017-08-30

**Authors:** Asier Carral-Menoyo, Verónica Ortiz-de-Elguea, Mikel Martinez-Nunes, Nuria Sotomayor, Esther Lete

**Affiliations:** Departamento de Química Orgánica II, Facultad de Ciencia y Tecnología, Universidad del País Vasco/Euskal Herriko Unibertsitatea UPV/EHU, Apdo. 644, 48080 Bilbao, Spain; asier.carral@ehu.eus (A.C.-M.); veronica.ortizdeelguea@ehu.eus (V.O.-d.-E.); mmartinez903@ikasle.ehu.eus (M.M.-N.)

**Keywords:** quinoline, synthesis, palladium, coupling, C–H alkenylation

## Abstract

Palladium-catalyzed dehydrogenative coupling is an efficient synthetic strategy for the construction of quinoline scaffolds, a privileged structure and prevalent motif in many natural and biologically active products, in particular in marine alkaloids. Thus, quinolines and 1,2-dihydroquinolines can be selectively obtained in moderate-to-good yields via intramolecular C–H alkenylation reactions, by choosing the reaction conditions. This methodology provides a direct method for the construction of this type of quinoline through an efficient and atom economical procedure, and constitutes significant advance over the existing procedures that require preactivated reaction partners.

## 1. Introduction

**Figure 1:**
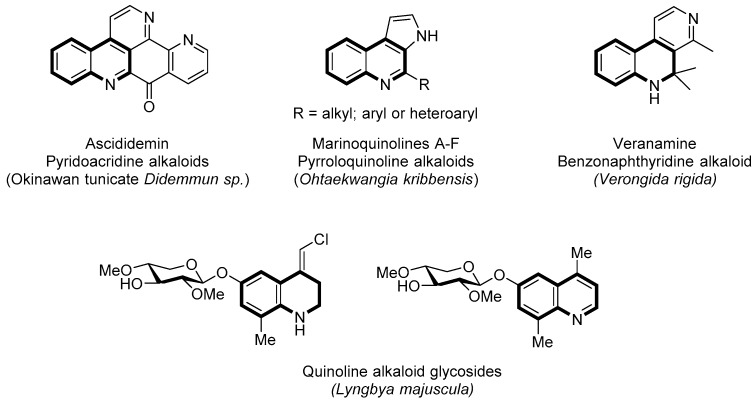
Selected bioactive marine alkaloids bearing a quinoline core.

Marine organisms are an increasingly important source of bioactive natural products, which in some cases have found application as pharmaceuticals (e.g., anticancer drugs) [1,2]. Quinoline core is a common structural motif among many marine alkaloids [3,4]. For example, the pyridoacridine family (e.g., ascididemin), a large class of marine alkaloids isolated from sessile organisms (sponges, corals, ascidians, bryozoans) [5,6], which display different types of biological activities, e.g., cytotoxicity, production of reactive oxygen species (ROS), and topoisomerase inhibition [7,8,9,10]. Marinoquinolines A–F are pyrroloquinolines isolated from gliding bacterium *Ohtaekwangia kribbensis* (Bacteroidetes) [11], whereas Veranamine is a benzonaphthyridine isolated from Florida sponges, namely, *Verongula rigida*, with significant antidepressant activity [12,13]. Besides, two quinoline alkaloid glycosides have been isolated in Puerto Rico from extracts of the cyanobacterium *Lyngbya majuscule* [14,15] (Figure 1). On the other hand, quinstatins, derived from dolastatin 10 (exceptionally anticancer drug contained in the sea hare *Dolabella auricularia*) by replacing the C-terminal dolaphenine (Doe) unit with a carefully designed quinoline, have been reported to be exceptional cancer cell growth inhibitors [16,17]. Cyanobacterial metabolites calothrixins have shown their potential as human DNA topisomerase I poisons for their cytotoxicity in cancer [18].

**Figure 2:**
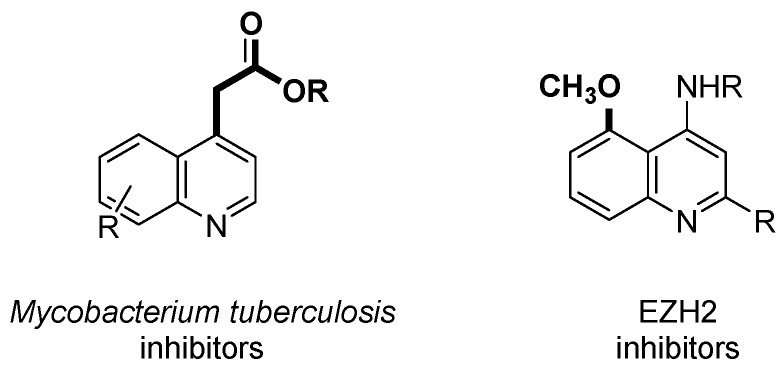
Characteristic structural features of some bioactive quinoline alkaloid analogues.

The outstanding biological activities of these marine alkaloids have attracted the attention of numerous research groups working toward the total synthesis of members of these natural products or analogues, either to get enough quantities or to establish structure-activity relationships for drug development. For example, it has been claimed that synthetic 4-alkylcarbonylmethyl or 4-alkoxycarbonylmethyl substituted quinolines show inhibitory activity against drug resistant *Mycobacterium tuberculosis* [19] and potent antimicrobial activity against *Helicobacter pylori* [20]. More recently, based on *SAR* studies, it has been demonstrated that the presence of a methoxyl group at C-5 position of the quinoline nucleus is structural feature common to a new class of Enhancer of Zeste Homologue 2 (EZH2) inhibitors, which could be useful for the treatment of several cancer types (lymphoma, colon, prostate, breast, and lung cancer) (Figure 2) [21].

**Figure 3:**
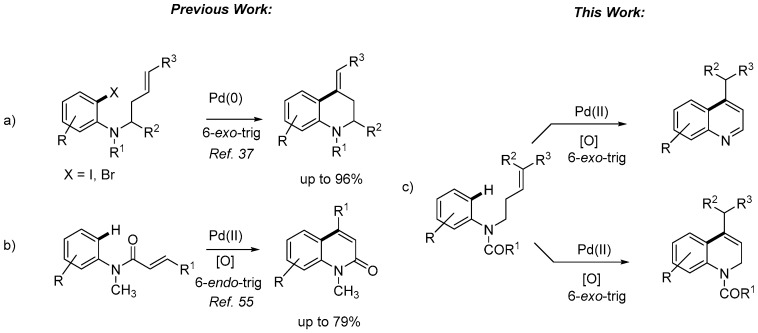
Palladium-catalyzed approaches to quinoline core.

Therefore, the development of new methodologies for the synthesis of quinolines and their dihydro/tetrahydro counterparts is well documented in the literature [22,23,24,25]. Among the several variants for synthesis of quinolines, the palladium-mediated cyclization processes [26,27,28,29] and, in particular, the intramolecular Mizoroki–Heck reaction [30,31,32,33] stand out as valuable synthetic protocols. In our research program on quinoline synthesis [34,35,36], we have reported [37] an effective protocol for the synthesis of 2-substituted 4-alkylidenetetrahydroquinoline derivatives, which employs a 6-*exo*-trig Mizoroki–Heck cyclization of *N*-alkenyl-substituted 2-haloanilines (Figure 3a). When non-substituted alkenes are used, the reaction can be directed towards the formation of an exocyclic or endocyclic carbon-carbon double bond, while 4-alkylidenetehtrahydroquinolines are obtained regioselectively with substituted alkenes. However, the Fujiwara-Moritani reaction offers notable advantages over traditional cross-coupling chemistry [38,39,40,41,42]. Reactions can be performed under air atmosphere even in aqueous media, and there is no need to prepare specifically functionalized cyclization precursors (i.e., *o*-haloanilines). This transformation, also known as oxidative Mizoroki–Heck reaction, consists in a direct coupling between two C–H centers (an aromatic C–H bond and an olefinic C–H bond), so it can be considered as either a C–H activation reaction or a C–H olefination. The reaction is catalyzed by Pd(II) and an external oxidant is required to regenerate the active catalytic species. Control of site selectivity is one of the most important challenges in this chemistry because organic molecules can contain a wide variety of C–H bonds [43]. The most common strategies for addressing this issue are the use of electronically activated substrates, directing groups [44,45,46] or ligands [47,48,49,50], which are able to coordinate to the metal center and deliver the catalyst to the targeted C–H bond. Intermolecular Fujiwara-Moritani reactions have received much attention recently, but examples of intramolecular variants are still scarce [51]. In particular, a number of reports have dealt with the construction of five membered rings via 5-*exo*-trig processes, and in many cases, the alkenylation of electron-rich heteroarenes is involved [52,53]. For example, intramolecular reaction of substituted *N*-phenylacrylamides catalyzed by Pd(II)-catalysts afforded oxindoles in moderate to good yields [54]. However, 6-*endo*-trig cyclizations are rare in Fujiwara-Moritani reactions and have been described only when the 5-*exo* process is blocked, not allowing the palladium hydride elimination. Nevertheless, we have been able to complete an unprecedented selective 6-*endo*-trig intramolecular C–H alkenylation of *N*-phenylacrylamides that led to 4-substituted quinolin-2[1*H*]-ones (Figure 3b) [55]. The adequate choice of the catalyst, oxidant, and experimental conditions allowed us to presumably change the steric and electronic properties around the metal center and direct the reaction to the β-position of the unsaturated moiety.

In this work we describe the use of the intramolecular C–H alkenylation reaction of *N*-buten-3-ylanilines for the synthesis of quinolines and dihydroquinolines, through 6-*exo*-trig cyclization processes. Thus, starting from the same precursors, conditions will be selected to favor nitrogen deprotection and oxidation to obtain the quinolines, or to avoid this over-oxidation and obtain 1,2-dihydroquinolines after isomerization of the double bond. The extension to different substitution patterns on the alkene or on the aromatic ring will also be described.

## 2. Results and Discussion

**Scheme 1:**
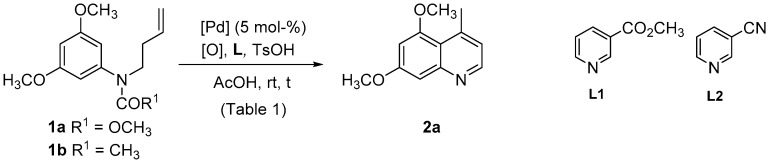
Pd(II)-catalyzed cyclization of **1a**,**b**.

The study began with the intramolecular Fujiwara-Moritani reaction of the *N*-substituted *N*-alkenylanilines **1a**,**b** (Scheme 1). To check the viability of the reaction, we first carried out a stoichiometric reaction with 1 equiv. of Pd(OAc)_2_ in acetic acid at reflux. In both cases, the *N*-protecting group was lost in the reaction, leading to quinoline **2a** in low yield (12%) through a 6-*exo*-trig cyclization, followed by isomerization of the double bond and aromatization. Pd(II)-catalytic conditions were subsequently investigated (Scheme 1, Table 1). Due to extensive decomposition observed at high temperature, Pd(II)-catalysis at room temperature was first studied. Pd(OAc)_2_ was initially used as Pd(II) source in acetic acid in the presence of *p*-toluenesulfonic acid, as these conditions have been found to be optimal for the cyclization of related amides [55]. Besides, among the wide range of oxidants that can be used for reoxidation of Pd(0) to Pd(II), we selected PhCO_3_*t*Bu [56], Cu(OAc)_2_ [57], *p*-benzoquinone [58] and *N*-fluoro-2,4,6-trimethylpyridinium triflate (F^+^) [59] for this preliminary screening. 

Thus, quinoline **2a** was obtained at room temperature with generally low yields, irrespective the oxidant, although the reactions were faster (5.5 h vs. 24 h) when acetate **1b** was used (Table 1, entries 1, 4, 7, 10, 13, 16). The use of ligands for palladium to increase the reactivity was also studied. In this context, pyridine ligands have been shown to enhance not only the reaction rate but also the site selectivity in Pd(II)-catalyzed reactions, and they have been used in intramolecular reactions, in combination with different oxidants [54,60,61,62]. We selected two pyridine ligands: ethyl nicotinate (**L1**) and 3-cyanopyridine (**L2**). However, the addition of these ligands for palladium had a detrimental effect in combination of all oxidants used, except for *p*-benzoquinone, for which a slight increase of the reactivity was observed (Table 1, entries 13 vs. 15 and 16 vs. 17). Various Pd(II) sources were also tested and Pd(dba)_2_ was revealed as a better catalyst than Pd(OAc)_2_ (Table 1, entries 19–22 vs. entries 1, 7, and 13). Finally, the best conditions implied the use of PdCl_2_(CH_3_CN)_2_ as Pd(II) source, and a combination of PhCO_3_*t*Bu and Cu(OAc)_2_ (5 mol %) as oxidative system, affording **2a** in moderate yields from both **1a** and **1b**, although the reaction was much faster with **1b** (Table 1, entries 23 and 25).

**Scheme 2:**
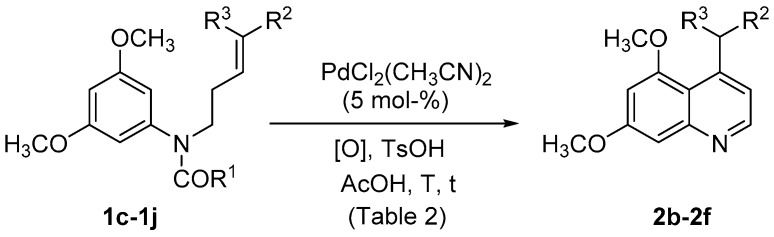
Synthesis of 4-substituted quinolines **2b**–**f**.

With these conditions in hand, the reaction was extended for the synthesis of quinolines with different substitution at C-4 (Scheme 2, Table 2). Thus, different electron withdrawing groups were incorporated in the alkene from **1a**,**b** through cross metathesis to obtain **1c**–**1j** (see Supplementary Materials). However, when phenylsulfonyl derivative **1d** was submitted to the previously optimized conditions, only low conversion (<10%) of the starting material was observed at rt (Table 2, entry 1), while decomposition was also observed when higher temperature was used (entry 2). The use of a more powerful oxidant, such as F^+^ and 70 °C were necessary to obtain 4-phenylsufonylmethylquinoline **2b** in moderate yield (Table 2, entry 4). The yield could be improved using the corresponding carbamate **1c** as starting material (Table 2, entry 5). Similarly, acetamide **1f** showed lower reactivity than the corresponding carbamate **1e**, since longer reaction time is needed to bring the reaction to completion (Table 2, entries 6 and 7), even using a higher catalyst loading. In both cases **2c** was obtained in moderate yields. When F^+^ was substituted for PhCO_3_*t*Bu as the oxidant, longer reaction times were required to obtain **2c** in lower yield (Table 2, entry 8). The scope of the reaction was further studied using only substrates possessing a carbamate-protecting group **1g**–**1j** due to their higher reactivity. Thus, the reaction proceeded efficiently for the synthesis of quinolines **2d**–**e** that bear different ester moieties (Table 2, entries 10–12), but failed when a trisubstituted olefin was used (Table 2, entry 9).

**Scheme 3:**
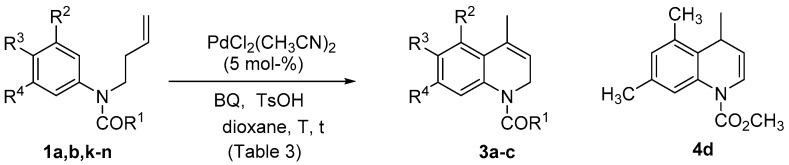
Synthesis of 4-substituted dihydroquinolines **3a**–**c** and **4d**.

As has been shown, the intramolecular Fujiwara-Moritani reaction allows the synthesis of 4-substituted quinolines through a 6-*exo*-trig cyclization followed by deprotection and aromatization. On the other hand, to apply this protocol for the synthesis of 1,2-dihydroisoquinolines, deprotection of the nitrogen atom should be avoided, thus preventing further oxidation. This would imply the use of milder reaction conditions, avoiding the use of acetic acid as solvent [63]. After some experimentation, we found that the cyclization could be efficiently performed in dioxane at room temperature, using *p*-benzoquinone as an oxidant in the presence of *p*-toluenesulfonic acid (Scheme 3). Under these reaction conditions, both protecting groups in **1a** and **1b** were stable, and dihydroquinolines **3a** and **3b** were obtained in good yields (Table 3, entries 1 and 3). Once again, the carbamate protected aniline **1a** was more reactive than **1b**, leading to a good yield of **3a** in shorter reaction time (7.5 h vs. 25 h). An increase of the reaction temperature to 70 °C led to a more efficient reaction, obtaining **3a** in high yield (89%) in only 10 min (Table 3, entry 2). Once again, the use of ligands **L1** and **L2** for palladium was detrimental, completely precluding the reaction. Then, the extension to other substitution patterns on the aromatic ring was studied. Interestingly, with a more electron rich aromatic ring (**1k**), the reaction was less efficient, and no cyclization was observed at room temperature after 24 h (Table 3, entry 6). An increase of the temperature was required to obtain **3c** in low yield (Table 3, entry 7), which could be improved increasing the catalyst loading (Table 3, entry 8), although an increase of the reaction time led to decomposition, lowering the isolated yield of **3c** (Table 3, entry 9). An electron-donor group *ortho* to the cyclization position appears to be necessary, as the reaction did not proceed at all for the 3,4-disubstituted substrates **1l** and **1m** (Table 3, entries 10 and 11). However, when weakly donor methyl groups are incorporated in 3,5-positions, the cyclization took place, but in this case isomerization of the double bond led to the formation of the 1,4-dihydroquinoline **4d**. 

**Scheme 4:**
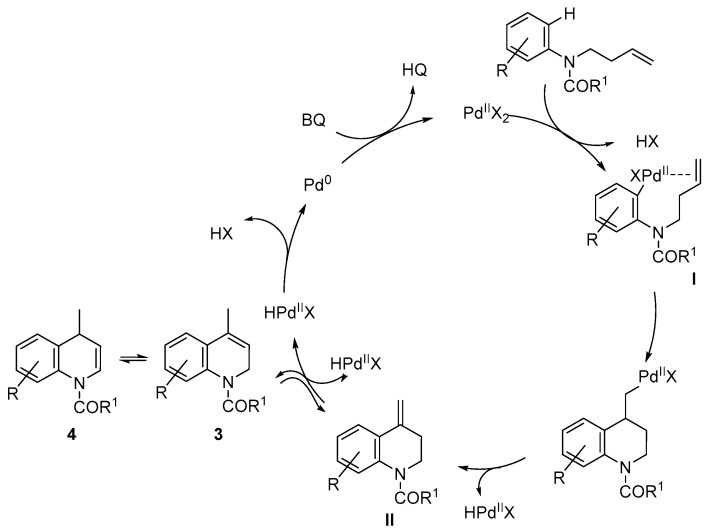
Mechanistic proposal.

These results are in agreement with the mechanistic proposal shown in Scheme 4. Thus, the first step would be the formation if the arylpalladium(II) intermediate **I**. Different mechanisms have been proposed for this palladation step, which is highly dependent on the substrate [50]. Thus, concerted metalation deprotonation (CMD), oxidative addition or electrophilic palladation mechanisms have been proposed. In this case, the electronic effects of the substituents on the aromatic ring would agree with an electrophilic palladation mechanism, favored by the electron-donor effects of the substituents on the aromatic ring. The subsequent migratory insertion followed by β-hydride elimination would form quinoline **II**, with an exocyclic double bond that would isomerize to the endocyclic position forming **3**, probably due to a higher thermodynamic stability. Pd(0) is finally oxidized to the Pd(II) active species by the oxidant. 

In conclusion, it has been shown that both quinolines and 1,2-dihydroquinolines can be selectively obtained in moderate to good yields via palladium(II)-catalyzed C–H alkenylation reactions, choosing the reaction conditions. Thus, when the reactions are carried out in acetic acid, deprotection and further oxidation leads to the one pot formation of 4-substituted quinolines **2a**–**f**. On the other hand, under milder reaction conditions, deprotection and over-oxidation can be avoided, leading to 1,2-dihydroquinolines **3a**–**c**. This procedure is complementary to the related Mizoroki-Heck reaction [37] that led to the formation of 4-methylidenetetrahydroquinolines, with exocyclic double bonds, with the advantage that this procedure does not require the prior functionalization of the substrates. However, the method is so far limited to the use of electron rich aromatic rings.

## 3. Materials and Methods

### 3.1. General Experimental Methods

Melting points were determined in unsealed capillary tubes and are uncorrected. IR spectra were obtained in film over NaCl pellets, or using an ATR. NMR spectra were recorded at 20–25 °C, at 300 MHz for ^1^H and 75.5 MHz for ^13^C or at 500 MHz for ^1^H and 125.7 MHz for ^13^C in CDCl_3_ solutions. Assignments of individual ^13^C and ^1^H resonances are supported by DEPT experiments and 2D correlation experiments (COSY, HSQCed or HMBC). Selective NOE or NOESY experiments were performed when necessary. Mass spectra were recorded under electron impact (EI) at 70 eV or under chemical ionization (CI) at 230 eV, or using Electrospray ionization (ESI^+^). Exact mass was obtained using a TOF detector. TLC was carried out with 0.2 mm thick silica gel plates. Visualization was accomplished by UV light. Flash column chromatography was performed on silica gel (230–400 mesh) or on alumina (70–230 mesh). All solvents used in reactions were anhydrous and purified according to standard procedures. All air- or moisture-sensitive reactions were performed under argon; the glassware was dried (130 °C) and purged with argon. Palladium catalysts were purchased from Sigma-Aldrich Química SL (Madrid, Spain), and were used without further purification: Pd(OAc)_2_ 98% purity, PdCl_2_(CH_3_CN)_2_, 99% purity. 

### 3.2. Synthesis of 4-Substituted Quinolines ***2***

*5,7-Dimethoxy-4-methylquinoline* (**2a**) (Table 1, entry 23). Over a solution of methyl but-3-en-1-yl(3,5-dimethoxyphenyl)carbamate (**1a**) (94.3 mg, 0.36 mmol) in AcOH (1.4 mL), PhCO_3_*t*Bu (0.09 mL, 0.43 mmol), Cu(OAc)_2_ (3.3 mg, 0.018 mmol), TsOH (64.8 mg, 0.36 mmol) and PdCl_2_(CH_3_CN)_2_ (4.7 mg, 0.018 mmol) were added. The mixture was stirred at room temperature for 24 h, and then the solvent was removed under vacuum. The residue was dissolved in AcOEt (5 mL) and it was washed with a 2 M aqueous solution of Na_2_CO_3_ (2 × 10 mL) and brine (2 × 10 mL). The aqueous phase was re-extracted with AcOEt (10 mL) and the combined organic extracts were dried (Na_2_SO_4_) and concentrated *in vacuo*. Flash column chromatography (silica gel, hexane/AcOEt 6/4) afforded **23** (40.3 mg, 56%) as an oil: IR (ATR) 1612 cm^−1^ (C=N); ^1^H NMR (CDCl_3_): δ 2.81 (s, 3H, CH_3_), 3.89 (s, 3H, OCH_3_), 3.92 (s, 3H, OCH_3_), 6.48 (d, *J* = 2.3 Hz, 1H, H_6_), 6.95 (d, *J* = 4.5 Hz, 1H, H_3_), 7.02 (d, *J* = 2.3 Hz, 1H, H_8_), 8.56 (d, *J* = 4.5 Hz, 1H, H_2_); ^13^C NMR (CDCl_3_): δ 24.3 (CH_3_), 55.5 (2 × OCH_3_), 98.6 (C_6_), 100.4 (C_3_), 116.8 (C_4a_), 121.4 (C_8_), 146.0 (C_4_), 150.0 (C_2_), 151.35 (C_8a_), 158.6 (C_5_), 160.4 (C_7_); MS (EI) *m*/*z* (rel intensity) 204.1 (M^+^ + 1, 13), 203.1 (M^+^, 100), 188 (28), 174.1 (12), 160.1 (11), 145 (14), 117 (11); HRMS (CI) calcd. for C_12_H_14_NO_2_ [MH^+^], 204.1025; found: 204.1025.

#### General Procedure for the Synthesis of 4-Substituted Quinolines **2b**–**f**

Over a solution of the corresponding butenyl aniline **1c**–**j** (1 mmol) in AcOH (11 mL), TsOH (1 mmol), *N*-fluoro-2,4,6-trimethylpyridinium triflate (F^+^) (1.2 mmol), Cu(OAc)_2_ (0.05 mmol) and PdCl_2_(CH_3_CN)_2_ (0.05 or 0.1 mmol) were added. The mixture was stirred at 70 °C for the specified time, and then the solvent was removed under vacuum. The residue was dissolved in AcOEt (5 mL) and it was washed with a 2 M aqueous solution of Na_2_SO_4_ (2 × 10 mL) and brine (2 × 10 mL). The aqueous phase was re-extracted with AcOEt (10 mL) and the combined organic extracts were dried (Na_2_SO_4_) and concentrated in vacuo. Flash column chromatography (silica gel, hexane/AcOEt) afforded the corresponding quinolines **2b**–**f** (Table 2).

*5,7-Dimethoxy-4-[(phenylsulfonyl)methyl]quinoline* (**2b**) (Table 2, entry 5). Prepared from **1c** (68.7 mg, 0.17 mmol), TsOH (32.7 mg, 0.17 mmol), F^+^ (59.4 mg, 0.20 mmol), Cu(OAc)_2_ (1.6 mg, 0.008 mmol) and PdCl_2_(CH_3_CN)_2_ (2.2 mg, 0.008 mmol) in AcOH (1.5 mL). The mixture was stirred at 70 °C for 24 h. After work-up and purification by flash column chromatography (silica gel, hexane/AcOEt 2/8), **2d** was obtained (35.3 mg, 61%) as an oil: IR (ATR) 1620 cm^−1^ (C=N), 1325 cm^−1^, 1135 cm^−1^ (R-SO_2_-R); ^1^H NMR (CDCl_3_): δ 3.71 (s, 3H, OCH_3_), 3.91 (s, 3H, OCH_3_), 5.22 (s, 2H, CH_2_), 6.36 (d, *J* = 2.4 Hz, 1H, H_6_), 7.01–7.05 (m, 2H, H_3_, H_8_), 7.28–7.37 (m, 2H, H_3′_, H_5′_), 7.46–7.53 (m, 3H, H_2′_, H_4′_, H_6′_), 8.69 (d, *J* = 4.5 Hz, 1H, H_2_); ^13^C NMR (CDCl_3_): δ 55.5 (OCH_3_), 55.6 (OCH_3_), 62.0 (CH_2_), 99.7 (C_6_), 101.1 (C_8_), 115.2 (C_4a_), 123.5 (C_3_), 128.6 (C_2′_, C_3′_, C_5′_, C_6′_), 133.6 (C_4′_), 134.1 (C_4_), 138.4 (C_1′_), 149.9 (C_2_), 151.7 (C_8a_), 156.7 (C_5_), 160.5 (C_7_); MS (ESI^+^) *m*/*z* (rel intensity) 345.1 (MH^+^ + 1, 19), 344.1 (MH^+^, 100); HRMS (ESI^+^) calcd. for C_18_H_18_NO_4_S [MH^+^], 344.0957; found: 344.0970.

*Methyl 2-(5,7-dimethoxyquinolin-4-yl)acetate* (**2c**) (Table 2, entry 6). Prepared from **1e** (93.3 mg, 0.29 mmol), TsOH (54.9 mg, 0.29 mmol), F^+^ (0.10 g, 0.35 mmol), Cu(OAc)_2_ (2.6 mg, 0.014 mmol) and PdCl_2_(CH_3_CN)_2_ (3.7 mg, 0.014 mmol) in AcOH (3.2 mL). The mixture was stirred at 70 °C for 19 h. After work-up and purification by flash column chromatography (silica gel, hexane/AcOEt 2/8), **2c** was obtained (40.8 mg, 54%) as a solid: mp (CH_2_Cl_2_) 77–80 °C; IR (ATR) 1735 cm^−1^(C=O); ^1^H NMR (CDCl_3_): δ 3.67 (s, 3H, COOCH_3_), 3.83 (s, 3H, OCH_3_), 3.91 (s, 3H, OCH_3_), 4.09 (s, 2H, CH_2_COOCH_3_), 6.49 (d, *J* = 2.2 Hz, 1H, H_6_), 6.95 (d, *J* = 4.4 Hz, 1H, H_3_), 7.02 (d, *J* = 2.2 Hz, 1H, H_8_), 8.67 (d, *J* = 4.4 Hz, 1H, H_2_); ^13^C NMR (CDCl_3_): δ 42.8 (CH_2_COOCH_3_), 51.8 (COOCH_3_), 55.2 (OCH_3_), 55.5 (OCH_3_), 99.0 (C_6_), 100.9 (C_8_), 116.0 (C_4a_), 122.2 (C_3_), 140.2 (C_4_), 150.4 (C_2_), 151.6 (C_8a_), 157.2 (C_5_), 160.5 (C_7_), 171.4 (CO); MS (EI) *m*/*z* (rel intensity) 262.1 (M^+^ + 1, 17), 261.2 (M^+^, 100), 229.1 (12), 202.1 (10), 186.1 (19), 172.1 (36); HRMS (ESI^+^) calcd. for C_14_H_16_NO_4_ [MH^+^], 262.1079; found: 262.1091.

*2,2,2-Trifluoroethyl 2-(5,7-dimethoxyquinolin-4-yl)acetate* (**2d**) (Table 2, entry 10). Prepared from **1h** (0.15 g, 0.39 mmol), TsOH (73.4 mg, 0.39 mmol), F^+^ (0.13 g, 0.46 mmol), Cu(OAc)_2_ (3.5 mg, 0.019 mmol) and PdCl_2_(CH_3_CN)_2_ (5.0 mg, 0.019 mmol) in AcOH (4.3 mL). The mixture was stirred at 70 °C for 21 h. After work-up and purification by flash column chromatography (silica gel, hexane/AcOEt 2/8), **6b** was obtained (58.3 mg, 46%) as a solid: mp (CH_2_Cl_2_) 95–97 °C; IR (ATR) 1745 cm^−1^(C=O); ^1^H NMR (CDCl_3_): δ 3.81 (s, 3H, OCH_3_), 3.90 (s, 3H, OCH_3_), 4.19 (s, 2H, CH_2_COOCH_2_), 4.48 (q, *J* = 8.5 Hz, 2H, COOCH_2_CF_3_), 6.50 (d, *J* = 2.2 Hz, 1H, H_6_), 6.95 (d, *J* = 4.4 Hz, 1H, H_3_), 7.06 (d, *J* = 2.2 Hz, 1H, H_8_), 8.67 (d, *J* = 4.4 Hz, 1H, H_2_); ^13^C NMR (CDCl_3_): δ 42.2 (CH_2_COOCH_2_), 55.3 (OCH_3_), 55.6 (OCH_3_), 60.5 (q, *J* = 36.6 Hz, COOCH_2_CF_3_), 99.3 (C_6_), 100.8 (C_8_), 115.8 (C_4a_), 122.3 (C_3_), 112.9 (q, *J* = 275.8 Hz, CF_3_), 139.2 (C_4_), 150.3 (C_2_), 151.5 (C_8a_), 157.0 (C_5_), 160.7 (C_7_), 169.5 (CO); MS (EI) *m*/*z* (rel intensity) 330.1 (M^+^ + 1, 17), 329.1 (M^+^, 100), 186 (21), 172.1 (27); HRMS (ESI^+^) calcd. for C_15_H_15_F_3_NO_4_ [MH^+^], 330.0953; found: 330.0956.

*Dodecyl 2-(5,7-dimethoxyquinolin-4-yl)acetate* (**2e**) (Table 2, entry 11). Prepared from **1i** (0.14 g, 0.29 mmol), TsOH (54.3 mg, 0.29 mmol), F^+^ (99.1 mg, 0.34 mmol), Cu(OAc)_2_ (3.1 mg, 0.017 mmol) and PdCl_2_(CH_3_CN)_2_ (4.4 mg, 0.017 mmol) in AcOH (3.2 mL). The mixture was stirred at 70 °C for 21 h. After work-up and purification by flash column chromatography (silica gel, hexane/AcOEt 3/7), **2f** was obtained (59.5 mg, 50%) as a solid: mp (CH_2_Cl_2_) 54–56 °C; IR (ATR) 1731 cm^−1^ (C=O); ^1^H NMR (CDCl_3_): δ 0.87 (t, *J* = 6.7 Hz, 3H, CH_3_), 1.10–1.35 (m, 18H, OCH_2_CH_2_(CH_2_)_9_CH_3_), 1.42–1.64 (m, 2H, CO_2_CH_2_CH_2_), 3.83 (s, 3H, OCH_3_), 3.91 (s, 3H, OCH_3_), 4.05 (t, *J* = 6.7 Hz, 2H, COOCH_2_), 4.09 (s, 2H, CH_2_COOCH_2_), 6.49 (d, *J* = 1.7 Hz, 1H, H_6_), 6.96 (d, *J* = 4.1 Hz, 1H, H_3_), 7.08 (d, *J* = 1.7 Hz, 1H, H_8_), 8.67 (br s, 1H, H_2_); ^13^C NMR (CDCl_3_): δ 14.1 (CH_3_), 22.7 (CH_3_CH_2_), 25.9 (COOCH_2_CH_2_CH_2_), 28.6, 29.2, 29.3, 29.5, 29.6, 29.7 (7 × CH_2_), 31.9 (CH_3_CH_2_CH_2_), 43.2 (CH_2_COOCH_2_), 55.2 (OCH_3_), 55.5 (OCH_3_), 64.9 (COOCH_2_), 99.0 (C_6_), 100.9 (C_8_), 116.0 (C_4a_), 122.2 (C_3_), 140.5 (C_4_), 150.4 (C_2_), 151.6 (C_8a_), 157.2 (C_5_), 160.5 (C_7_), 171.0 (CO); MS (EI) *m*/*z* (rel intensity) 416.3 (M^+^ + 1, 8), 415.3 (M^+^, 30), 386.3 (24), 372.2 (23), 358.2 (21), 344.2 (18), 330.2 (18), 316.2 (21), 302.1 (21), 248.1 (12), 247.1 (10), 204.1 (11), 203.1 (72), 202.1 (25), 189.1 (10), 188.1 (100), 173.1 (18), 172.1 (56), 129 (10), 57.1 (12), 55.1 (19); HRMS (ESI^+^) calcd. for C_25_H_38_NO_4_ [MH^+^], 416.2801; found: 416.2809.

*Benzyl 2-(5,7-dimethoxyquinolin-4-yl)acetate* (**2f**) (Table 2, entry 12). Prepared from **1j** (0.11 g, 0.28 mmol), TsOH (53.9 mg, 0.28 mmol), F^+^ (98.3 mg, 0.34 mmol), Cu(OAc)_2_ (2.6 mg, 0.014 mmol) and PdCl_2_(CH_3_CN)_2_ (3.7 mg, 0.014 mmol) in AcOH (3.1 mL). The mixture was stirred at 70 °C for 21 h. After work-up and purification by flash column chromatography (silica gel, hexane/AcOEt 2/8), **2g** was obtained (58.9 mg, 62%) as an oil: IR (ATR) 1735 cm^−1^ (C=O); ^1^H NMR (CDCl_3_): δ 3.54 (s, 3H, OCH_3_), 3.92 (s, 3H, OCH_3_), 4.12 (s, 2H, CH_2_COOCH_2_Ph), 5.12 (s, 2H, CH_2_Ph) 6.41 (d, *J* = 2.3 Hz, 1H, H_6_), 6.97 (d, *J* = 4.5 Hz, 1H, H_3_), 7.06 (d, *J* = 2.3 Hz, 1H, H_8_), 7.14-7.49 (m, 5H, Ph), 8.67 (d, *J* = 4.5 Hz, 1H, H_2_); ^13^C NMR (CDCl_3_): δ 43.1 (CH_2_COOCH_2_Ph), 54.9 (OCH_3_), 55.5 (OCH_3_), 66.3 (CH_2_Ph), 99.0 (C_6_), 100.8 (C_8_), 116.0 (C_4a_), 122.3 (C_3_), 128.2 (C_2′_, C_6′_), 128.5 (C_3′_, C_4′_, C_5′_), 136.0 (C_1′_), 140.2 (C_4_), 150.4 (C_2_), 151.6 (C_8a_), 157.1 (C_5_), 160.5 (C_7_), 170.7 (CO); MS (EI) *m*/*z* (rel intensity) 338.1 (M^+^ + 1, 20), 337.1 (M^+^, 92), 172.1 (47), 91.1 (100); HRMS (ESI^+^) calcd. for C_20_H_20_NO_4_ [MH^+^], 338.1392; found: 338.1418.

### 3.3. Synthesis of 4-Substituted Dihydroquinolines ***3*** and ***4***

#### General Procedure

Over a solution of the corresponding *N*-substituted but-3-en-1-ylaniline **1a**,**b**,**k**–**n** (1 mmol) in dioxane (66.7 mL), TsOH (1 mmol), *p*-benzoquinone (1 mmol) and PdCl_2_(CH_3_CN)_2_ (0.05 mmol) were added. The reaction mixture was stirred for the specified time at room temperature or at 70 °C. Afterwards, water was added to quench the reaction and the mixture was extracted with CH_2_Cl_2_ (3 × 10 mL). The combined organic extracts were washed with brine (10 mL), dried (Na_2_SO_4_) and concentrated in vacuo. Flash column chromatography (silica gel, hexane/AcOEt) afforded the corresponding 1,2-dihydroquinoles **3a**–**c** or 1,4-dihydroquinoline **4d**.

*Methyl 5,7-dimethoxy-4-methylquinoline-1(2H)-carboxylate* (**3a**) (Table 3, entry 2). Prepared from carbamate **1a** (106.5 mg, 0.40 mmol), TsOH (77.5 mg, 0.40 mmol), *p*-benzoquinone (44.1 mg, 0.40 mmol) and PdCl_2_(CH_3_CN)_2_ (5.3 mg, 0.020 mmol) in dioxane (31 mL). The reaction mixture was stirred for 10 min at 70 °C and after work-up and purification by flash column chromatography (silica gel, hexane/AcOEt 8/2), **3a** was obtained (94.2 mg, 89%) as an oil: IR (ATR) 1706 cm^−1^ (C=O); ^1^H NMR (CDCl_3_): δ 2.16 (d, *J* = 1.3 Hz, 3H, CH_3_) 3.77 (s, 3H, COOCH_3_), 3.79 (s, 3H, OCH_3_), 3.81 (s, 3H, OCH_3_), 4.10–4.15 (m, 2H, CH_2_), 5.64 (td, *J* = 4.8, 1.3 Hz, 1H, H_3_), 6.29 (d, *J* = 2.4 Hz, 1H, H_6_), 6.80 (br s, 1H, H_8_); ^13^C NMR (CDCl_3_): δ 21.9 (CH_3_), 42.7 (C_2_), 53.0 (COOCH_3_), 55.4 (OCH_3_), 55.5 (OCH_3_), 96.1 (C_6_), 101.4 (C_8_), 113.6 (C_4a_), 119.9 (C_3_), 132.7 (C_4_), 139.4 (C_8a_), 154.4 (CO), 158.0 (C_5_), 159.0 (C_7_); MS (EI) *m*/*z* (rel intensity) 264.1 (M^+^ + 1, 13), 263.1 (M^+^, 79), 249.1 (14), 248.1 (100), 205.1 (11), 204.1 (84), 203.1 (50), 189.1 (29), 188.1 (24), 174.1 (13), 160.1 (16), 146.1 (11), 145.1 (11), 130.1 (10), 117.1 (10); HRMS (ESI^+^) calcd. for C_14_H_18_NO_4_ [MH^+^], 264.1236; found: 264.1259.

*1-[5,7-Dimethoxy-4-methylquinolin-1(2H)-yl]ethanone* (**3b**) (Table 3, entry 3). Prepared from acetamide **1b** (0.12 g, 0.46 mmol), TsOH (88.1 mg, 0.46 mmol), *p*-benzoquinone (50.1 mg, 0.46 mmol) and PdCl_2_(CH_3_CN)_2_ (6.0 mg, 0.023 mmol) in dioxane (31 mL). The reaction mixture was stirred for 25 h at rt, and after work-up and purification by flash column chromatography (silica gel, hexane/AcOEt 6/4), **3b** was obtained (71.2 mg, 62%) as a mixture of rotamers in a 6:4 ratio and as an oil: IR (ATR) 1699 (C=O); ^1^H NMR (CDCl_3_): δ 2.19 (s, 3H, CH_3_, both rotamers), 2.23 (s, 3H, COCH_3_, both rotamers), 3.83 (s, 6H, 2 × OCH_3_, both rotamers), 4.21 (br s, 2H, CH_2_, both rotamers), 5.71 (br s, 6.36, major rotamer: 2H, H_6_, H_8_; minor rotamer: 1H, H_6_), 6.75 (br s, 1H, H_8_, minor rotamer); ^13^C NMR (CDCl_3_): δ 21.9 (CH_3_, both rotamers), 22.7 (COCH_3_, both rotamers), 40.9 (C_2_, both rotamers), 55.4 (OCH_3_, both rotamers), 55.5 (OCH_3_, both rotamers), 96.5 (C_6_, both rotamers), 102.3 (C_8_, major rotamer), 114.1 (C_4a_, both rotamers), 116.1 (C_8_, minor rotamer), 122.2 (C_3_, both rotamers), 132.2 (C_4_, both rotamers), 139.9 (C_8a_, minor rotamer), 149.84 (C_8a_, major rotamer), 157.8 (C_5_, both rotamers), 158.8 (C_7_, both rotamers), 169.7 (CO, both rotamers); MS (EI) *m*/*z* (rel intensity) 248.1 (M^+^ + 1, 3), 247.1 (M^+^, 18), 205.1 (15), 204.1 (100), 203.1 (10), 190.1 (28), 189.1 (19); HRMS (ESI^+^) calcd. for C_14_H_18_NO_3_ [MH^+^], 248.1287, found: 248.1294.

*Methyl 5,6,7-trimethoxy-4-methylquinoline-1(2H)-carboxylate* (**3c**) (Table 3, entry 8). Prepared from carbamate **1k** (0.107 g, 0.36 mmol), TsOH (70 mg, 0.36 mmol), *p*-benzoquinone (40 mg, 0.36 mmol) and PdCl_2_(CN)_2_ (9.5 mg, 0.036 mmol) in dioxane (31 mL). The reaction mixture was stirred for 2 h at 70 °C, and after work-up and purification by flash column chromatography (silica gel, hexane/AcOEt 6/4) affording **3c** as an oil (42 mg, 40%): IR (ATR) 1685 cm^−1^ (C=O); ^1^H NMR (CDCl_3_): δ 2.18 (s, 3H, CH_3_), 3.77 (s, 3H, CO_2_CH_3_), 3.82 (s, 3H, OCH_3_), 3.84 (s, 3H, OCH_3_), 3.86 (s, 3H, OCH_3_), 4.11 (brs, 2H, NCH_2_), 5.69 (brs, 1H, H_3_), 6.98 (br s, 1H, H_8_); ^13^C NMR (CDCl_3_): δ 21.3 (CH_3_), 42.6 (C_2_), 53.0 (CO_2_*C*H_3_), 56.0 (OCH_3_), 60.8 (OCH_3_), 61.1 (OCH_3_), 104.3 (C_8_), 117.8 (C_4a_), 121.2 (C_3_), 132.2 (C_4_), 133.5 (C_8a_), 139.8 (C_6_), 150.8 (C_5_), 151.9 (C_7_), 154.5 (CO); MS (EI) *m*/*z* (rel intensity): 293 (M^+^, 69), 278 (100), 262 (8), 234 (40), 218 (24), 207 (27), 204 (21), 190 (17), 176 (13); HRMS (ESI^+^) calcd. for C_15_H_20_NO_5_ [M + H]^+^, 294.1341; found: 294.1361.

*Methyl 4,5,7-trimethylquinoline-1(4H)-carboxylate* (**4d**) (Table 3, entry 12). Prepared from carbamate **1n** (110.0 mg, 0.47 mmol), TsOH (91.1 mg, 0.47 mmol), *p*-benzoquinone (52.0 mg, 0.47 mmol) and PdCl_2_(CN)_2_ (12.4 mg, 0.047 mmol) in dioxane (31 mL). The reaction mixture was stirred for 24 h at 70 °C, and after work-up and purification by flash column chromatography (silica gel, hexane/AcOEt 9:1) affording the product **4d** as an oil (34.4 mg, 32%): IR (ATR) 1730 cm^−1^ (C=O); ^1^H NMR (CDCl_3_): δ 1.16 (d, *J* = 6.9 Hz, 3H, CHCH_3_), 2.29 (s, 3H, CH_3_), 2.31 (s, 3H, CH_3_), 3.44–3.52 (m, 1H, H_4_), 3.87 (s, 3H, OCH_3_), 5.48 (t, *J* = 6.9 Hz, 1H, H_3_), 6.84 (s, 1H, H_6_), 6.95 (d, *J* = 6.9 Hz, 1H, H_2_), 7.62 (s, 1H, H_8_); ^13^C NMR (CDCl_3_): δ 18.7 (CH*C*H_3_), 21.2 (CH_3_), 29.0 (C_4_), 53.1 (OCH_3_), 116.3 (C_3_), 120.3 (C_8_), 125.9 (C_6_), 127.8 (C_4a_), 129.4 (C_2_), 134.6 (C_8a_), 135.3 (C_7_), 136.1 (C_5_), 153.3 (CO); MS (EI) *m*/*z* (rel intensity): 231 (M^+^, 65), 215 (100), 199 (8), 171 (84), 156 (21); HRMS (ESI^+^) calcd. for C_14_H_18_NO_2_ [M + H]^+^, 232.1338; found: 232.1344.

## Figures and Tables

**Table 1:** Optimization of cyclization conditions for **1a**,**b**.

Entry	Substrate	[Pd]	[O]	L ^(a)^	t (h)	2a Yield (%)
1	**1a**	Pd(OAc)_2_	PhCO_3_*t*Bu ^(b)^	-	24	19
2	**1a**	Pd(OAc)_2_	PhCO_3_*t*Bu ^(b)^	**L1**	24	11
3	**1a**	Pd(OAc)_2_	PhCO_3_*t*Bu ^(b)^	**L2**	24	5
4	**1b**	Pd(OAc)_2_	PhCO_3_*t*Bu ^(b)^	-	5.5	12
5	**1b**	Pd(OAc)_2_	PhCO_3_*t*Bu ^(b)^	**L1**	5.5	11
6	**1b**	Pd(OAc)_2_	PhCO_3_*t*Bu ^(b)^	**L2**	5.5	9
7	**1a**	Pd(OAc)_2_	Cu(OAc)_2_^(c)^	-	24	36
8	**1a**	Pd(OAc)_2_	Cu(OAc)_2_^(c)^	**L1**	24	35
9	**1a**	Pd(OAc)_2_	Cu(OAc)_2_^(c)^	**L2**	24	14
10	**1b**	Pd(OAc)_2_	Cu(OAc)_2_^(c)^	-	5.5	20
11	**1b**	Pd(OAc)_2_	Cu(OAc)_2_^(c)^	**L1**	5.5	nr
12	**1b**	Pd(OAc)_2_	Cu(OAc)_2_^(c)^	**L2**	5.5	nr
13	**1a**	Pd(OAc)_2_	*p*-BQ ^(c)^	-	24	4
14	**1a**	Pd(OAc)_2_	*p*-BQ ^(c)^	**L1**	14	5
15	**1a**	Pd(OAc)_2_	*p*-BQ ^(c)^	**L2**	24	24
16	**1b**	Pd(OAc)_2_	*p*-BQ ^(c)^	-	24	10
17	**1b**	Pd(OAc)_2_	*p*-BQ ^(c)^	**L1**	24	19
18	**1b**	Pd(OAc)_2_	*p*-BQ ^(c)^	**L2**	24	8
19	**1a**	Pd(dba)_2_	PhCO_3_*t*Bu ^(b)^	-	24	28
20	**1a**	Pd(dba)_2_	Cu(OAc)_2_^(c)^	-	24	54
21	**1a**	Pd(dba)_2_	*p*-BQ ^(c)^	-	24	15
22	**1a**	Pd(dba)_2_	F^+ (c)^	-	24	18
**23**	**1a**	**PdCl_2_(CH_3_CN)_2_**	**PhCO_3_*t*Bu ^(b)^**	**-**	**24**	**56**
24	**1a**	PdCl_2_(CH_3_CN)_2_	Cu(OAc)_2_^(c)^	-	48	20
**25**	**1b**	**PdCl_2_(CH_3_CN)_2_**	**PhCO_3_*t*Bu ^(b)^**	**-**	**3.5**	**55**
26	**1b**	PdCl_2_(CH_3_CN)_2_	F^+ (c)^	-	24	27

^(a)^ 20 mol %; ^(b)^ 1.2 equiv. Cu(OAc)_2_ (5 mol %) was used as co-oxidant; ^(c)^ 1 equiv.

**Table 2:** Extension to substituted alkenes. Synthesis of quinolines **2b**–**f**.

Entry	Substrate	R^1^	R^2^	R^3^	[O]	T (°C)	t (h)	2	Yield (%)
1	**1d**	CH_3_	SO_2_Ph	H	PhCO_3_*t*Bu ^(a)^	rt	24	**2b**	^(c)^
2	**1d**	CH_3_	SO_2_Ph	H	PhCO_3_*t*Bu ^(a)^	70	24	**2b**	^(c),(d)^
3	**1d**	CH_3_	SO_2_Ph	H	F^+ (a)^	rt	24	**2b**	^(c),(d)^
4	**1d**	CH_3_	SO_2_Ph	H	F^+ (a)^	70	24	**2b**	44
5	**1c**	OCH_3_	SO_2_Ph	H	F^+ (a)^	70	24	**2b**	61
6	**1e**	OCH_3_	CO_2_CH_3_	H	F^+ (a)^	70	19	**2c**	54
7	**1f**	CH_3_	CO_2_CH_3_	H	F^+ (a),(b)^	70	41	**2c**	54
8	**1f**	CH_3_	CO_2_CH_3_	H	PhCO_3_*t*Bu ^(a)^	70	47	**2c**	32
9	**1g**	OCH_3_	CO_2_CH_3_	CH_3_	F^+ (a)^	70	21	**-**	^(d)^
10	**1h**	OCH_3_	CO_2_CH_2_CF_3_	H	F^+ (a)^	70	21	**2d**	46
11	**1i**	OCH_3_	CO_2_(CH_2_)_11_CH_3_	H	F^+ (a)^	70	21	**2e**	50
12	**1j**	OCH_3_	CO_2_CH_2_Ph	H	F^+ (a)^	70	21	**2f**	62

^(a)^ 1.2 equiv. Cu(OAc)_2_ (5 mol %) was used as co-oxidant; ^(b)^ Additional 5 mol % of catalyst was added; ^(c)^ Minor formation of product observed by ^1^H NMR; ^(d)^ Decomposition.

**Table 3:** Synthesis of 4-substituted dihydroquinolines **3a**–**c** and **4d**.

Entry	Substrate	R^1^	R^2^	R^3^	R^4^	T (°C)	t (h/min)	Product	Yield (%)
1	**1a**	OCH_3_	OCH_3_	H	OCH_3_	rt	7.5 h	**3a**	74
2	**1a**	OCH_3_	OCH_3_	H	OCH_3_	70	10 min	**3a**	89
3	**1b**	CH_3_	OCH_3_	H	OCH_3_	rt	25 h	**3b**	62
4	**1b**	CH_3_	OCH_3_	H	OCH_3_	rt ^(a)^	23 h	**-**	^(c)^
5	**1b**	CH_3_	OCH_3_	H	OCH_3_	rt ^(b)^	23 h	**-**	^(c)^
6	**1k**	OCH_3_	OCH_3_	OCH_3_	OCH_3_	rt	24 h	**-**	^(c)^
7	**1k**	OCH_3_	OCH_3_	OCH_3_	OCH_3_	70	2 h	**3c**	33
8	**1k**	OCH_3_	OCH_3_	OCH_3_	OCH_3_	70 ^(d)^	2 h	**3c**	40
9	**1k**	OCH_3_	OCH_3_	OCH_3_	OCH_3_	70 ^(d)^	7 h	**3c**	11
10	**1l**	OCH_3_	H	OCH_2_O		70 ^(d)^	24 h	**-**	^(c)^
11	**1m**	OCH_3_	H	OCH_3_	OCH_3_	70 ^(d)^	24 h	**-**	^(c)^
12	**1n**	OCH_3_	CH_3_	H	CH_3_	70 ^(d)^	24 h	**4d**	32

^(a)^ **L1** (5 mol %) was added; ^(b)^ **L2** (5 mol %) was added; ^(c)^ No reaction. Recovered starting material; ^(d)^ 10 mol % of catalyst was used.

## Supplementary Materials

The following are available online at www.mdpi.com/1660-3397/15/9/276/s1: Preparation procedures for the substrates **1a**–**n**. Copies of ^1^H and ^13^C NMR spectra of compounds **1**–**4.**
