# Supplementary material for: Palladium-Catalyzed Dehydrogenative Coupling: An Efficient Synthetic Strategy for the Construction of the Quinoline Core
Source: Mar Drugs. 2017 Aug 30;15(9):276. doi: 10.3390/md15090276 (PMC5618415; doi:10.3390/md15090276)

Supplementary Material

Palladium-catalyzed dehydrogenative coupling: an efficient synthetic strategy for the construction of the quinoline core

Asier Carral-Menoyo,[1] Verónica Ortiz-de-Elguea,[1] Mikel Martinez-Nunes,[1] Nuria Sotomayor[1,*] and Esther Lete[1,*]

[1] Departamento de Química Orgánica II, Facultad de Ciencia y Tecnología, Universidad del País Vasco / Euskal Herriko Unibertsitatea (UPV/EHU), Apdo. 644. 48080 Bilbao (Spain).

Table of contents

1. Synthesis of substituted anilines **1a-n.** S2

1.1. General procedure for the akylation of protected anilines **S5**. Synthesis of **1a,b** and **1k-n** S2

1.2. General procedure for the cross metathesis reaction. Synthesis of **1c-j.** S4

2. Copies of ^{1}H and ^{13}C NMR spectra of compounds described S8

1. Synthesis of substituted anilines 1a-n

Substrates **1a-b**, and **1k-n** were prepared by alkylation of the corresponding protected aniline with buten-3-yl tosylate. Subsequent cross metathesis catalyzed by Grubbs second generation catalyst provided the substituted alkenes **1c-j.**

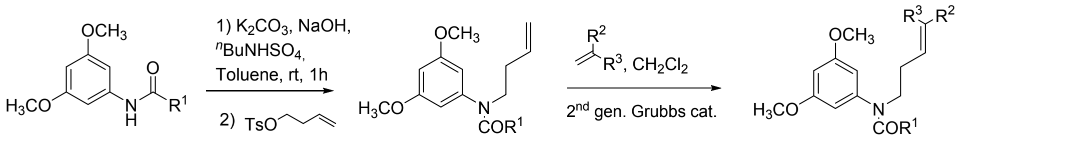

S5a R^1= OCH_3
S5b R^1 = CH_3

1a R^1 = OCH_3
1b R^1 = CH_3

1c R^1 = OCH_3, R^2 = SO_2Ph, R^3 = H
1d R^1 = CH_3, R^2 = SO_2Ph, R^3 = H
1e R^1 = OCH_3, R^2 = CO_2CH_3, R^3 = H
1f R^1 = CH_3, R^2 = CO_2CH_3, R^3 = H
1g R^1 = OCH_3, R^2 = CO_2CH_3, R^3 = CH_3
1h R^1 = OCH_3, R^2 = $CO_2CH_2CF_3$, R^3 = H
1i R^1 = OCH_3, R^2 = $CO_2(CH_2)_{11}CH_3$, R^3 = H
1j R^1 = OCH_3, R^2 = CO_2Bn, R^3 = H

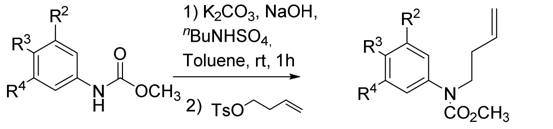

S5k R^2 = R^3 = R^4 = OCH_3
S5l R^2= H, R^3 - R^4 = $-OCH_2O-$
S5m R^2= H, R^3 = R^4 = OCH_3
S5n R^2= CH_3, R^3 = H, R^4 = CH_3

1k R^2 = R^3 = R^4 = OCH_3
1l R^2= H, R^3 - R^4 = $-OCH_2O-$
1m R^2= H, R^3 = R^4 = OCH_3
1n R^2= CH_3, R^3 = H, R^4 = CH_3

Scheme S1. Preparation of substrates **1a-n**

1.1. General procedure for the akylation of protected anilines S5. Synthesis of 1a,b and 1k-n. Over a solution of the corresponding protected aniline **S5** (1 mmol) in toluene (3 mL), anhydrous K_2CO_3 (1 mmol), powdered NaOH (4 mmol) and $^nBu_4NHSO_4$ (0.05 mmol) were added. The mixture was stirred for 1 h at room temperature and then, it was heated at 80 ºC for 15 min. Afterwards, a solution of but-3-enyl-4-methylbenzenesulfonate (1.6 mmol) in toluene (0.7 mL) was added, and the reaction was heated at 80 ºC for the indicated time. The mixture was allowed to cool down to room temperature, and a 1 M aqueous solution of HCl (25 mL) was added. The organic layer was separated and the aqueous layer was extracted with Et_2O (3 × 20 mL). The combined organic extracts were dried (Na_2SO_4) and concentrated *in vacuo*. Flash column chromatography (silica gel, hexane/AcOEt) afforded the corresponding *N*-substituted but-3-en-1-ylanilines **1a,b** and **1k-n.**

Methyl but-3-en-1-yl(3,5-dimethoxyphenyl)carbamate (1a). Prepared from methyl (3,5-dimethoxyphenyl)carbamate **S5a** (2.05 g, 9.72 mmol), anhydrous K_2CO_3 (1.34 g, 9.72 mmol), powdered NaOH (1.55 g, 38.9 mmol) and $^nBu_4NHSO_4$ (0.16 g, 0.49 mmol) in toluene (29.2 mL). The mixture was stirred for 1 h at room temperature and then, it was heated at 80 ºC for 15 min. Afterwards, a solution of but-3-enyl-4-methylbenzenesulfonate **1c** (3.51 g, 15.6 mmol) in toluene (11 mL) was added, and the reaction was heated at 80 ºC for 28 h. After work-up, the crude reaction product was purified by flash column chromatography (silica gel, hexane/AcOEt 8/2), affording **1a** (2.40 g, 93%) as an oil: IR (ATR) 1706 cm^{-1} (C=O); ^1H NMR ($CDCl_3$): δ 2.17-2.31 (m, 2H, $NCH_2C\underline{H_2}$), 3.51-3.78 (m, 11H, $COOC\underline{H_3}$, $NC\underline{H_2}CH_2$, 2 × $OC\underline{H_3}$), 4.82-5.11 (m, 2H, $CH=C\underline{H_2}$), 5.69 (ddt, *J* = 17.0, 10.2, 6.8 Hz, 1H, $C\underline{H}=CH_2$), 6.30 (s, 3H, H_2, H_4, H_6); ^{13}C NMR ($CDCl_3$): δ 32.7 ($NCH_2\underline{C}H_2$), 49.6 ($N\underline{C}H_2CH_2$), 52.6 ($COO\underline{C}H_3$), 55.2 (2 ×

$O\underline{C}H_3$), 98.7 (C_4), 105.9 (C_2, C_6), 116.6 ($CH{=}\underline{C}H_2$), 135.2 ($\underline{C}H{=}CH_2$), 143.3 (C_1), 155.8 ($\underline{C}O$), 160.8 (C_3, C_5); MS (EI) *m/z* (rel intensity) 265.1 (M^+, 25), 225.1 (13), 224.1 (100), 211.1 (12), 180.1 (23), 165.1 (22), 152.1 (39), 137.1 (10); HRMS (CI) calcd. for $C_{14}H_{20}NO_4$ [MH^+], 266.1396; found: 266.1392.

***N*-(But-3-en-1-yl)-*N*-(3,5-dimethoxyphenyl)acetamide (1b).** Prepared from *N*-(3,5-dimethoxyphenyl)acetamide **S5b** (2.05 g, 10.5 mmol), anhydrous K_2CO_3 (1.45 g, 10.5 mmol), powdered NaOH (1.68 g, 42.0 mmol) and $^nBu_4NHSO_4$ (0.18 g, 0.53 mmol) in toluene (31.5 mL). The mixture was stirred for 1 h at room temperature and then, it was heated at 80 ºC for 15 min. Afterwards, a solution of but-3-enyl-4-methylbenzenesulfonate (3.80 g, 16.8 mmol) in toluene (9.5 mL) was added, and the reaction was heated at 80 ºC for 4 h. After work-up, the crude reaction product was purified by flash column chromatography (silica gel, hexane/AcOEt 6/4), affording **1b** (1.73 g, 66%) as an oil: IR (ATR)1656 cm^{-1} (C=O); ^1H NMR ($CDCl_3$): δ 1.80 (s, 3H, $COC\underline{H_3}$), 2.18-2.32 (m, 2H, $NCH_2C\underline{H_2}$), 3.65-3.72 (m, 2H, $NC\underline{H_2}CH_2$), 3.74 (s, 6H, 2 × OCH_3), 4.95-5.08 (m, 2H, $CH{=}C\underline{H_2}$), 5.69 (ddt, *J* = 17.0, 10.2, 6.7 Hz, 1H, $C\underline{H}{=}CH_2$), 6.25 (d, *J* = 2.2 Hz, 2H, H_2, H_6), 6.37 (t, *J* = 2.2 Hz, 1H, H_4); ^{13}C NMR ($CDCl_3$): δ 22.4 ($CO\underline{C}H_3$), 32.2 ($NCH_2\underline{C}H_2$), 47.8 ($N\underline{C}H_2CH_2$), 55.3 (2 × OCH_3), 99.4 (C_4), 106.4 (C_2, C_6), 116.4 ($CH{=}\underline{C}H_2$), 135.3 ($\underline{C}H{=}CH_2$), 144.5 (C_1), 161.3 (C_3, C_5), 169.8 ($\underline{C}O$); MS (EI) *m/z* (rel intensity) 249.1 (M^+, 6), 195.1 (12), 167.1 (10), 166.1 (100); HRMS (CI) calcd. for $C_{14}H_{20}NO_3$ [MH^+], 250.1443; found: 250.1455.

Methyl but-3-en-1-yl(3,4,5-trimethoxyphenyl)carbamate (1k). Prepared from carbamate **S5k** (0.40 g, 1.66 mmol), anhydrous K_2CO_3 (0.24 g, 1.66 mmol), powder NaOH (0.27 g, 6.66 mmol) and *n*-Bu_4NHSO_4 (0.029 g, 0.083 mmol) in toluene (20 mL). The mixture was stirred for 1 h at room temperature and then heated at 80 °C for 15 min. Afterwards but-3-enyl 4-methylbenzenesulfonate (0.60 g, 2.65 mmol) in toluene (3 mL) was added and the reaction mixture was heated at 80 °C for 28 h. After work-up, the crude reaction product was purified by flash column chromatography (silica gel, hexane/AcOEt 60:40) affording **1k** as an oil (0.29 g, 59 %): IR (ATR) 1700 cm^{-1} (C=O); ^1H NMR ($CDCl_3$): δ 2.26-2.37 (m, 2H, $NCH_2C\underline{H_2}$), 3.64-3.76 (m, 5H, $COOCH_3$, NCH_2), 3.83 (s, 3H, OCH_3), 3.85 (s, 3H, OCH_3), 3.87 (s, 3H, OCH_3), 5.04-5.11 (m, 2H, $NCH_2CH_2CH{=}C\underline{H_2}$), 5.78 (ddt, *J* = 17.1 Hz, 10.3 Hz, 6.8 Hz, 1 H, $NCH_2CH_2C\underline{H}{=}CH_2$), 6.41 (s, 2H, H_2, H_6); ^{13}C NMR ($CDCl_3$): δ (ppm) = 32.7 (NCH_2CH_2), 49.9 ($CO_2\underline{C}H_3$), 52.8 (NCH_2), 56.1 ($C_3O\underline{C}H_3$, $C_5O\underline{C}H_3$), 60.7 ($C_4O\underline{C}H_3$), 105.1 (C_2, C_6), 116.7 ($CH{=}\underline{C}H_2$), 135.2 (C_1), 136.8 (C_4), 137.3 ($\underline{C}H{=}CH_2$), 153.2 (C_3,C_5), 156.0 (NCO); MS (EI) *m/z* (rel intensity): 295 (M^+, 71), 280 (19), 254 (100), 226 (23), 195 (46), 182 (24), 180 (51), 167 (13); HRMS (ESI^+) calcd. for $C_{15}H_{22}NO_5$ $[M+H]^+$, 296.1498; found: 296.1509.

Methyl benzo[d][1,3]dioxol-5-yl(but-3-en-1-yl)carbamate (1l). Prepared from carbamate **S5l** (0.72 g, 3.72 mmol), anhydrous K_2CO_3 (0.49 g, 3.72 mmol), powder NaOH (0.57 g, 14.88 mmol) and*n*-Bu_4NHSO_4 (0.060 g, 0.19 mmol) in toluene (45 mL).). The mixture was stirred for 1 h at room temperature and then heated at 80 °C for 15 min. Afterwards but-3-enyl 4-methylbenzenesulfonate (1.26 g, 5.55 mmol) in toluene (5 mL) was added, and the reaction mixture was heated at 80 °C for 28 h. After work-up, the crude reaction product was purified by flash column chromatography (silica gel, hexane/AcOEt 70:30) affording **1l** as an oil (0.79 g, 85 %): IR (ATR) 1700 cm^{-1} (C=O); ^1H NMR ($CDCl_3$): δ 2.20-2.34 (m, 2H, $NCH_2C\underline{H_2}$), 3.60-3.70 (m, 5H, $COOCH_3$, NCH_2), 4.98-5.09 (m, 2H, $NCH_2CH_2CH{=}C\underline{H_2}$), 5.73 (ddt, *J* = 17.0 Hz, 10.2 Hz, 6.8 Hz, 1 H, $NCH_2CH_2C\underline{H}{=}CH_2$), 5.95 (s, 2H, OCH_2O), 6.59-6.66 (m, 2H, H_2, H_5), 6.75 (d, *J* = 8.1 Hz, 1H, H_6); ^{13}C NMR ($CDCl_3$): δ 32.6 (NCH_2CH_2), 50.0 ($CO_2\underline{C}H_3$), 52.8 (NCH_2), 101.5 (OCH_2O), 108.1 (C_2), 109.0 (C_5), 116.8 (C_6), 120.8 ($CH{=}\underline{C}H_2$), 135.1 (C_1), 135.5 ($\underline{C}H{=}CH_2$), 146.3 (C_4), 147.8 (C_3), 156.2 (NCO); MS (EI) *m/z* (rel intensity): 249 (M^+, 40), 209 (11), 208 (100), 176 (9), 164 (21), 149 (65), 136 (27), 106 (13); HRMS (ESI^+) calcd. for $C_{13}H_{16}NO_4$ $[M+H]^+$, 250.1079; found: 250.1092.

Methyl but-3-en-1-yl(3,4-dimethoxyphenyl)carbamate (1m). Prepared from carbamate **S5m** (0.63 g, 2.96 mmol), anhydrous K_2CO_3 (0.42 g, 2.96 mmol), powder NaOH (0.48 g, 11.85 mmol) and *n*-Bu_4NHSO_4 (0.051 g, 0.15 mmol) in toluene (35 mL). The mixture was stirred for 1 h at room temperature and then, it was heated at 80 ºC for 15 min. Afterwards, a solution of but-3-enyl-4-methylbenzenesulfonate (1.072 g, 4.74 mmol) in toluene (4.5 mL) was added, and the reaction mixture was heated at 80 °C for 28 h. After work-up, the crude reaction product was purified by flash column chromatography (silica gel, hexane/AcOEt 70:30) affording **1m** as an oil (0.71 g, 91 %): IR (ATR) 1700 cm^{-1} (C=O); ^{1}H NMR ($CDCl_3$): δ 2.23-2.30 (m, 2H, $NCH_2C\underline{H_2}$), 3.62-3.71 (m, 5H, $COOCH_3$, NCH_2), 3.82 (s, 3H, OCH_3), 3.84 (s, 3H, OCH_3), 4.97-5.07 (m, 2H, $NCH_2CH_2CH{=}C\underline{H_2}$), 5.73 (ddt, *J* = 17.0 Hz, 10.2 Hz, 6.8 Hz, 1H, $NCH_2CH_2C\underline{H}{=}CH_2$), 6.62-6.75 (m, 2H, H_2, H_5), 6.80 (d, *J* = 8.4 Hz, 1H, H_6); ^{13}C NMR ($CDCl_3$): δ 32.6 ($NCH_2\underline{C}H_2$), 49.9 ($CO_2\underline{C}H_3$), 52.7 (NCH_2), 55.8 (OCH_3), 111.0 (C_2), 111.4 (C_5), 116.6 (C_6), 119.6 ($CH{=}\underline{C}H_2$), 134.5 (C_1), 135.2 ($\underline{C}H{=}CH_2$), 147.8 (C_4), 149.0 (C_3), 156.1 (NCO); MS (EI) m/z (rel intensity): 265 (M^+, 47), 225 (13), 224 (100), 192 (15), 180 (11), 165 (50), 152 (39), 150 (31); HRMS (ESI^+) calcd. for $C_{14}H_{20}NO_4$ $[M+H]^+$; 266.1396; found: 266.1393.

Methyl but-3-en-1-yl(3,5-dimethylphenyl)carbamate (1n). Prepared from carbamate **S5n** (0.87 g, 4.88 mmol), anhydrous K_2CO_3 (0.70 g, 4.88 mmol), powder NaOH (0.80 g, 19.54 mmol) and *n*-Bu_4NHSO_4 (0.085 g, 0.25 mmol) in toluene (58.5 mL). The mixture was stirred for 1 h at room temperature and then, it was heated at 80 ºC for 15 min. Afterwards, a solution of but-3-enyl-4-methylbenzenesulfonate (1.77 g, 7.82 mmol) in toluene (7 mL) was added, and the reaction mixture was heated at 80 °C for 28 h. After work-up, the crude reaction product was purified by flash column chromatography (silica gel, hexane/AcOEt 70:30) affording **1n** as an oil (0.87 g, 76 %): IR (ATR) 1700 cm^{-1} (C=O); ^{1}H NMR ($CDCl_3$): δ 2.19-2.39 (m, 8H, $NCH_2C\underline{H_2}$, 2 x CH_3), 3.62-3.76 (m, 5H, $COOCH_3$, NCH_2), 4.99-5.10 (m, 2H, $NCH_2CH_2CH{=}C\underline{H_2}$), 5.76 (ddt, *J* = 17.1 Hz, 10.4 Hz, 6.8 Hz, 1 H, $NCH_2CH_2C\underline{H}{=}CH_2$), 6.80 (s, 2H, H_2, H_6), 6.90 (s, 1H, H_4); ^{13}C NMR ($CDCl_3$): δ 21.2 (CH_3), 32.7 ($NCH_2\underline{C}H_2$), 49.9 (CO_2CH_3), 52.8 (NCH_2), 116.6 ($CH{=}\underline{C}H_2$), 125.2 (C_2, C_6), 128.6 (C_4), 135.2 ($\underline{C}H{=}CH_2$), 138.6 (C_3, C_5), 141.5 (C_1), 156.2 (NCO); MS (EI) *m/z* (rel intensity): 233 (M^+, 12), 192 (100), 148 (28), 133 (26), 121 (18), 105 (25); HRMS (ESI^+) calcd. for $C_{14}H_{20}NO_2$ $[M+H]^+$, 234.1494; found: 234.1503.

1.2. General procedure for the cross metathesis reaction. Synthesis of 1c-j. Over a solution of the corresponding *N*-but-3-en-1-ylaniline **1a-b** (1 mmol) in dry CH_2Cl_2 (29 mL) under argon atmosphere, the corresponding acrylate (10 or 20 mmol) or phenyl vinyl sulfone (2.5 mmol) was added. The mixture was stirred, and then, a solution of 2nd generation Grubbs catalyst (0.05 mmol) in dry CH_2Cl_2 (8 mL) was added *via canula*. The mixture was heated under reflux for 72 or 24 h, and every 24 h additional amounts of the catalyst (5 mol%) were added. Afterwards, the mixture was allowed to cool down to room temperature and the solvent was removed under reduced pressure. Flash column chromatography (silica gel, hexane/AcOEt 6/4) afforded the corresponding products **1c-j**.

(*E*)-Methyl (3,5-dimethoxyphenyl)[4-(phenylsulfonyl)but-3-en-1-yl]carbamate (1c). Prepared from carbamate **1a** (0.12 g, 0.45 mmol) and phenyl vinyl sulfone (0.18 g, 1.09 mmol) in dry CH_2Cl_2 (13 mL), as well as a solution of 2nd generation Grubbs catalyst (19.1 mg, 0.023 mmol) in dry CH_2Cl_2 (3.7 mL). The reaction mixture was heated under reflux for 72 h and additional amounts of the catalyst (19.1 mg, 0.023 mmol) were added every 24 h. After purification by flash column chromatography, **1c** was obtained (0.13 g, 71%) as an oil: IR (ATR) 1702 cm^{-1} (C=O), 1229 cm^{-1}, 1157 cm^{-1} (R-SO_2-R); ^{1}H NMR ($CDCl_3$): δ 2.43 − 2.54 (m, 2H, $NCH_2C\underline{H_2}$), 3.65 (s, 3H, $COOCH_3$), 3.70 − 3.86 (m, 8H, $NC\underline{H_2}CH_2$, 2 × OCH_3), 6.25 (d, *J* = 2.2 Hz, 2H, H_2, H_6), 6.30 − 6.42 (m, 2H, H_4, $CH{=}C\underline{H}$-SO_2Ph), 6.91 (dt, *J* = 15.2, 6.9 Hz, 1H, $C\underline{H}{=}CH$-SO_2Ph),

7.46 – 7.66 (m, 3H, $H_{2'}$, $H_{4'}$, $H_{6'}$), 7.76 – 7.91 (m, 2H, $H_{3'}$, $H_{5'}$); ^{13}C NMR ($CDCl_3$): δ 30.4 ($NCH_2\underline{C}H_2$), 48.4 ($N\underline{C}H_2CH_2$), 53.1 ($COO\underline{CH_3}$), 55.4 (2 × OCH_3), 99.1 (C_4), 105.7 (C_2, C_6), 127.6 ($C_{2'}$, $C_{6'}$), 129.3 ($C_{3'}$, $C_{5'}$), 132.2 ($C_{4'}$), 133.4 ($\underline{C}H=CH-SO_2Ph$), 140.4 ($C_{1'}$), 142.7 (C_1), 143.1 ($CH=\underline{C}H-SO_2Ph$), 155.8 (CO), 161.0 (C_3, C_5); MS (ESI^+) *m/z* (rel intensity) 407.1 (MH^+ + 1, 20), 406.1 (MH^+, 100), 374.1 (1); HRMS (ESI^+) calcd. for $C_{20}H_{24}NO_6S$ [MH^+], 406.1324; found: 406.1326.

***(E)*-*N*-(3,5-dimethoxyphenyl)-*N*-[4-(phenylsulphonyl)but-3-en-1-yl]acetamide (1d).** Prepared from acetamide **1b** (0.15 g, 0.59 mmol) and phenyl vinyl sulfone (0.25 g, 1.47 mmol) in dry CH_2Cl_2 (17 mL), as well as a solution of 2nd generation Grubbs catalyst (25.0 mg, 0.029 mmol) in dry CH_2Cl_2 (4.7 mL). The reaction mixture was heated under reflux for 72 h and additional amounts of the catalyst (25.0 mg, 0.029 mmol) were added every 24 h. After purification by flash column chromatography, **1d** was obtained (0.18 g, 77%) as an oil: IR (ATR) 1652 cm^{-1} (C=O), 1311, 1143 cm^{-1} ($R-SO_2-R$); ^1H NMR ($CDCl_3$): δ (1.84 (s, 3H, $CO\underline{H_3}$), 2.43-2.57 (m, 2H, $NCH_2C\underline{H_2}$), 3.71 − 3.89 (m, 8H, $NC\underline{H_2}CH_2$, 2 × $OC\underline{H_3}$), 6.23 (d, *J* = 2.2 Hz, 2H, H_2, H_6), 6.31 − 6.49 (m, 2H, H_4, $CH=C\underline{H}-SO_2Ph$), 6.92 (dt, *J* = 14.8, 6.8 Hz, 1H, $C\underline{H}=CH-SO_2Ph$), 7.43 – 7.67 (m, 3H, $H_{2'}$, $H_{4'}$, $H_{6'}$), 7.76 – 7.95 (m, 2H, $H_{3'}$, $H_{5'}$); ^{13}C NMR ($CDCl_3$): δ 22.5 ($CO\underline{C}H_3$), 29.9 ($NCH_2\underline{C}H_2$), 46.7 ($N\underline{C}H_2CH_2$), 55.5 (2 × $O\underline{C}H_3$), 99.8 (C_4), 106.2 (C_2, C_6), 127.6 ($C_{2'}$, $C_{6'}$), 129.3 ($C_{3'}$, $C_{5'}$), 132.2 ($C_{4'}$), 133.4 ($\underline{C}H=CH-SO_2Ph$), 140.5 ($C_{1'}$), 143.4 ($CH=\underline{C}H-SO_2Ph$), 144.2 (C_1), 161.6 (C_3, C_5), 170.5 ($\underline{C}O$); MS (CI) *m/z* (rel intensity) 391.1 (MH^++ 1, 20), 390.1 (MH^+, 79), 348.1 (22), 250.1 (26), 249.1 (16), 248.1 (67), 247.1 (14), 232.1 (16), 231.1 (13), 208.1 (12), 206.1 (21), 197.1 (17), 196.1 (99), 195.1 (100), 180.1 (11), 166.1 (29), 153.1 (15), 125 (17), 111 (42), 110 (19); HRMS (CI) calcd. for $C_{20}H_{24}NO_5S$ [MH^+], 390.1375; found: 390.1378.

***(E)*-Methyl 5-[(3,5-dimethoxyphenyl)(methoxycarbonyl)amino]pent-2-enoate (1e).** Prepared from methyl carbamate **1a** (1.08 g, 4.09 mmol) and methyl acrylate (7.37 mL, 81.8 mmol) in dry CH_2Cl_2 (118 mL), as well as a solution of 2nd generation Grubbs catalyst (0.17 g, 0.20 mmol) in dry CH_2Cl_2 (33 mL). The reaction mixture was heated under reflux for 72 h and additional amounts of the catalyst (0.17 g, 0.20 mmol) were added every 24 h. After purification by flash column chromatography, **1e** was obtained (1.22 g, 88%) as a solid: mp (CH_2Cl_2) 68-70 ºC; IR (ATR) 1727 cm^{-1} (CO_2CH_3), 1691 cm^{-1} (NCO_2CH_3); ^1H NMR ($CDCl_3$): δ 2.40-2.52 (m, 2H, $NCH_2C\underline{H_2}$), 3.62-3.91 (m, 14H, 2 × $OC\underline{H_3}$, $COOC\underline{H_3}$, $NCOOC\underline{H_3}$, $NC\underline{H_2}CH_2$), 5.82-5.91 (m, 1H, $CO-C\underline{H}=CH$), 6.32 (d, *J* = 2.1 Hz, 2H, H_2, H_6), 6.37 (t, *J* = 2.1 Hz, 1H, H_4), 6.88 (dt, *J* = 15.7, 7.3 Hz, 1H, $CO-CH=C\underline{H}$); ^{13}C NMR ($CDCl_3$): δ 31.2 ($NCH_2\underline{C}H_2$), 48.9 ($N\underline{C}H_2CH_2$), 51.5 ($COO\underline{C}H_3$), 53.0 ($COO\underline{C}H_3$), 55.4 (2 × $O\underline{C}H_3$), 99.1 (C_4), 106.8 (C_2, C_6), 122.8 ($CO-\underline{C}H=CH$), 143.1 (C_1), 145.4 ($CO-CH=\underline{C}H$), 155.8 ($N\underline{C}OOCH_3$), 161.0 (C_3, C_5), 166.6 ($\underline{C}OOCH_3$); MS (EI) *m/z* (rel intensity) 323.1 (M^+, 11), 250.1 (18), 225.1 (13), 224.1 (100), 211.1 (18), 180.1 (21), 165.1 (19), 152.1 (36); HRMS (ESI) calcd. for $C_{16}H_{22}NO_6$ [MH^+], 324.1447; found: 324.1458.

***(E)*-Methyl 5-[*N*-(3,5-dimethoxyphenyl)acetamido]pent-2-enoate (1f).** Prepared from acetamide **1b** (0.15 g, 0.59 mmol) and methyl acrylate (1.05 mL, 11.7 mmol) in dry CH_2Cl_2 (17 mL), as well as a solution of 2nd generation Grubbs catalyst (24.8 mg, 0.029 mmol) in dry CH_2Cl_2 (4.7 mL). The reaction mixture was heated under reflux for 72 h and additional amounts of the catalyst (24.8 mg, 0.029 mmol) were added every 24 h. After purification by flash column chromatography, **1f** was obtained (0.17 g, 91%) as a solid: mp (CH_2Cl_2) 63-65 ºC; IR (ATR) 1724 cm^{-1} (CO_2CH_3), 1656 cm^{-1} ($COCH_3$); ^1H NMR ($CDCl_3$): δ 1.87 (s, 3H, $C\underline{H_3}$), 2.40-2.53 (m, 2H, $NCH_2C\underline{H_2}$), 3.60-3.86 (m, 11H, 2 × $OC\underline{H_3}$, $COOC\underline{H_3}$, $NC\underline{H_2}CH_2$), 5.84 (d, *J* = 15.7 Hz, 1H, $CO-C\underline{H}=CH$), 6.27 (d, *J* = 2.1 Hz, 2H, H_2, H_6), 6.43 (t, *J* = 2.1 Hz, 1H, H_4), 6.87 (dt, *J* = 15.7, 7.7 Hz, 1H, $CO-CH=C\underline{H}$); ^{13}C NMR ($CDCl_3$): δ 22.4 ($CO\underline{C}H_3$), 31.7 ($NCH_2\underline{C}H_2$), 47.2 ($N\underline{C}H_2CH_2$), 51.3 ($COO\underline{C}H_3$), 55.4 (2 × $O\underline{C}H_3$), 99.6 (C_4), 106.3 (C_2, C_6), 122.6 ($CO-\underline{C}H=CH$), 144.3 (C_1), 145.6 (CO-

CH=$\underline{C}$H), 161.4 (C_3, C_5), 166.5 ($\underline{C}OOCH_3$), 170.1 ($N\underline{C}OCH_3$); MS (EI) *m/z* (rel intensity) 307.2 (M^+, 12), 234.1 (34), 208.1 (10), 195.1 (15), 167.2 (24), 166.2 (100), 153.1 (12); HRMS (ESI) calcd. for $C_{16}H_{22}NO_5$ [MH^+], 308.1498; found: 308.1507.

***(E)*-Methyl 5-[(3,5-dimethoxyphenyl)(methoxycarbonyl)amino]-2-methylpent-2-enoate (1g).** Prepared from carbamate **1a** (0.36 g, 1.36 mmol) and methyl methacrylate (2.91 mL, 27.3 mmol) in dry CH_2Cl_2 (39.4 mL), as well as a solution of 2nd generation Grubbs catalyst (57.9 mg, 0.068 mmol) in dry CH_2Cl_2 (11 mL). The reaction mixture was heated under reflux for 72 h and additional amounts of the catalyst (57.9 mg, 0.068 mmol) were added every 24 h. After purification by flash column chromatography, **1g** was obtained (0.42 g, 92%) as an oil: IR (ATR) 1706 cm^{-1} (C=O); ^1H NMR ($CDCl_3$): δ 1.76 (s, 3H, $C\underline{H_3}C$=CH), 2.36-2.46 (m, 2H, $NCH_2C\underline{H_2}$), 3.56-3.80 (m, 14H, 2 × $OC\underline{H_3}$, $NCOOC\underline{H_3}$, $COOC\underline{H_3}$, $NC\underline{H_2}CH_2$), 6.29 (d, *J* = 1.5 Hz, 2H, H_2, H_6), 6.31-6.35 (m, 1H, H_4), 6.61-6.69 (m, 1H, CO-C(CH_3)=C$\underline{H}$); ^{13}C NMR ($CDCl_3$): δ 12.5 ($\underline{C}H_3$), 27.8 ($NCH_2\underline{C}H_2$), 49.2 ($N\underline{C}H_2CH_2$), 51.7 ($COO\underline{C}H_3$), 52.9 ($COO\underline{C}H_3$), 55.3 (2 × $O\underline{C}H_3$), 98.9 (C_4), 106.7 (C_2, C_6), 129.5 (CO-$\underline{C}$(CH_3)=CH), 138.2 (CO-C(CH_3)=$\underline{C}$H), 143.2 (C_1), 155.8 ($N\underline{C}OOCH_3$), 160.9 (C_3, C_5), 168.2 ($\underline{C}OOCH_3$); MS (EI) *m/z* (rel intensity) 338.2 (M^+ + 1, 2), 337.2 (M^+, 10), 305.1 (13), 225.1 (16), 224.2 (100), 211.1 (16), 180.1 (24), 165.1 (18), 152.1 (37); HRMS (ESI) calcd. for $C_{17}H_{24}NO_6$ [MH^+], 338.1604; found: 338.1604.

***(E)*-2,2,2-Trifluoroethyl 5-[(3,5-dimethoxyphenyl)(methoxycarbonyl)amino]pent-2-enoate (1h).** Prepared from carbamate **1a** (0.30 g, 1.12 mmol) and 2,2,2-trifluoroethyl acrylate (1.42 mL, 11.2 mmol) in dry CH_2Cl_2 (32.4 mL), as well as a solution of 2nd generation Grubbs catalyst (47.6 mg, 0.056 mmol) in dry CH_2Cl_2 (9 mL). The reaction mixture was heated under reflux for 72 h and additional amounts of the catalyst (47.6 mg, 0.056 mmol) were added every 24 h. After purification by flash column chromatography, **1h** was obtained (0.41 g, 94%) as an oil: IR (ATR) 1739 cm^{-1} (CO_2CH_2), 1701 cm^{-1} (NCO_2CH_3); ^1H NMR ($CDCl_3$): δ 2.45-2.57 (m, 2H, $NCH_2C\underline{H_2}$), 3.56-3.91 (m, 11H, 2 × $OC\underline{H_3}$, $NCOOC\underline{H_3}$, $NC\underline{H_2}CH_2$), 4.49 (q, *J* = 8.5 Hz, 2H, $CO_2C\underline{H_2}CF_3$), 5.90 (d, *J* = 15.7 Hz, 1H, CO-C$\underline{H}$=CH), 6.31 (d, *J* = 1.9 Hz, 2H, H_2, H_6), 6.36-6.39 (m, 1H, H_4), 7.01 (dt, *J* = 15.7, 7.1 Hz, 1H, CO-CH=C$\underline{H}$); ^{13}C NMR ($CDCl_3$): δ 31.4 ($NCH_2\underline{C}H_2$), 48.7 ($N\underline{C}H_2CH_2$), 52.9 ($COO\underline{C}H_3$), 55.3 (2 × $O\underline{C}H_3$), 60.0 (q, *J* = 36.6 Hz, $COO\underline{C}H_2CF_3$), 99.0 (C_4), 106.8 (C_2, C_6), 121.2 (CO-$\underline{C}$H=CH), 123.0 (q, *J* = 275.8 Hz, $\underline{C}F_3$), 143.0 (C_1), 148.3 (CO-CH=$\underline{C}$H), 155.8 ($N\underline{C}OOCH_3$), 160.9 (C_3, C_5), 164.2 ($\underline{C}OOCH_2$); MS (EI) *m/z* (rel intensity) 392.2 (M^+ + 1, 4), 391.2 (M^+, 20), 250.1 (19), 225.1 (14), 224.2 (100), 211.1 (15), 180.1 (23), 165.1 (19), 152.1 (35); HRMS (ESI) Calcd. for $C_{17}H_{21}NO_6F_3$ [MH^+], 392.1321; found: 392.1329.

***(E)*-Dodecyl 5-[(3,5-dimethoxyphenyl)(methoxycarbonyl)amino]pent-2-enoate (1i).** Prepared from carbamate **1a** (0.29 g, 1.10 mmol) and lauryl acrylate (3.0 mL, 11.0 mmol) in dry CH_2Cl_2 (31.9 mL), as well as a solution of 2nd generation Grubbs catalyst (46.8 mg, 0.055 mmol) in dry CH_2Cl_2 (8.9 mL). The reaction mixture was heated under reflux for 72 h and additional amounts of the catalyst (46.8 mg, 0.055 mmol) were added every 24 h. After purification by flash column chromatography, **1i** was obtained (0.53 g, quant.) as an oil: IR (ATR) 1713 cm^{-1} (C=O); ^1H NMR ($CDCl_3$): δ 0.82 (t, *J* = 6.6 Hz, 3H, $C\underline{H_3}$), 1.15-1.33 (m, 18H, $OCH_2CH_2(C\underline{H_2})_9CH_3$), 1.52-1.64 (m, 2H, $CO_2CH_2C\underline{H_2}$), 2.36-2.46 (m, 2H, $NCH_2C\underline{H_2}$), 3.53-3.80 (m, 11H, 2 × $OC\underline{H_3}$, $NCOOC\underline{H_3}$, $NC\underline{H_2}CH_2$), 4.04 (t, *J* = 6.7 Hz, 2H, $CO_2C\underline{H_2}$), 5.79 (d, *J* = 15.7 Hz, 1H, CO-C$\underline{H}$=CH), 6.28 (d, *J* = 1.8 Hz, 2H, H_2, H_6), 6.29-6.33 (m, 1H, H_4), 6.67-6.96 (m, 1H, CO-CH=C$\underline{H}$); ^{13}C NMR ($CDCl_3$): δ 14.7 ($\underline{C}H_3$), 22.7 ($CH_3\underline{C}H_2$), 25.9 ($COOCH_2CH_2\underline{C}H_2$), 28.6, 29.2, 29.3, 29.5, 29.6, 29.7, (7 × $\underline{C}H_2$), 31.2 ($NCH_2\underline{C}H_2$), 31.9 ($CH_3CH_2\underline{C}H_2$), 48.9 ($N\underline{C}H_2CH_2$), 52.9 ($COO\underline{C}H_3$), 55.3 (2 × $O\underline{C}H_3$), 64.4 ($COO\underline{C}H_2$), 99.0 (C_4), 106.8 (C_2, C_6), 123.3 (CO-$\underline{C}$H=CH), 143.1 (C_1), 145.0 (CO-CH=$\underline{C}$H), 155.8

(N$\underline{C}$OOCH$_3$), 161.0 (C$_3$, C$_5$), 166.2 ($\underline{C}$OOCH$_2$); MS (ESI) *m/z* (rel intensity) 479.3 (MH$^+$ + 1, 25), 478.3 (MH$^+$, 100); HRMS (ESI$^+$) calcd. for $C_{27}H_{44}NO_6$ [MH$^+$], 478.3169; found: 478.3171.

***(E)*-Benzyl 5-[(3,5-dimethoxyphenyl)(methoxycarbonyl)amino]pent-2-enoate (1j).** Prepared from carbamate **1a** (0.24 g, 0.92 mmol) and benzyl acrylate (1.40 mL, 9.2 mmol) in dry CH_2Cl_2 (26.5 mL), as well as a solution of 2nd generation Grubbs catalyst (38.9 mg, 0.046 mmol) in dry CH_2Cl_2 (7.4 mL). The reaction mixture was heated under reflux for 24 h and after purification by flash column chromatography, **1j** was obtained (0.32 g, 86%) as an oil: IR (ATR)1706 cm^{-1} (C=O); ^{1}H NMR (CDCl$_3$): δ 2.43-2.53 (m, 2H, NCH$_2$C$\underline{H_2}$), 3.64-3.81 (m, 11H, NC$\underline{H_2}$CH$_2$, NCOOC$\underline{H_3}$, 2 × OC$\underline{H_3}$), 5.16 (s, 2H, C$\underline{H_2}$Ph), 5.86-5.94 (m, 1H, CO-C$\underline{H}$=CH), 6.33 (d, *J* = 2.2 Hz, 2H, H$_2$, H$_6$), 6.39 (t, *J* = 2.2 Hz, 1H, H$_4$), 6.94 (dt, *J* = 15.7, 7.1 Hz, 1H, CO-CH=C$\underline{H}$), 7.22 – 7.40 (m, 5H, Ph); ^{13}C NMR (CDCl$_3$): δ 31.3 (NCH$_2$$\underline{C}H_2$), 48.9 (N$\underline{C}H_2CH_2$), 53.0 (COO$\underline{C}H_3$), 55.6 (2 × O$\underline{C}H_3$), 66.1 ($\underline{C}H_2$Ph), 99.1 (C$_4$), 106.8 (C$_2$, C$_6$), 122.9 (CO-$\underline{C}$H=CH), 128.1 (C$_{2'}$,C$_{6'}$), 128.2 (C$_{4'}$), 128.6 (C$_{3'}$,C$_{5'}$), 136.1 (C$_{1'}$), 143.1 (C$_1$), 145.9 (CO-CH=$\underline{C}$H), 155.8 (N$\underline{C}$OOCH$_3$), 161.0 (C$_3$, C$_5$), 165.9 ($\underline{C}$OOCH$_2$); MS (EI) *m/z* (rel intensity) 400.2 (M$^+$ + 1, 2), 399.2 (M$^+$, 7), 308.1 (24), 250.1 (16), 248.1 (19), 225.1 (13), 224.1 (100), 211.1 (13), 180.1 (25), 165.1 (19), 152,1 (37), 91.1 (49); HRMS (ESI$^+$) calcd. for $C_{22}H_{26}NO_6$ [MH$^+$], 400.1760; found: 400.1760.

5. Copies of ^{1}H and ^{13}C NMR spectra of compounds described.

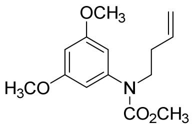

1a

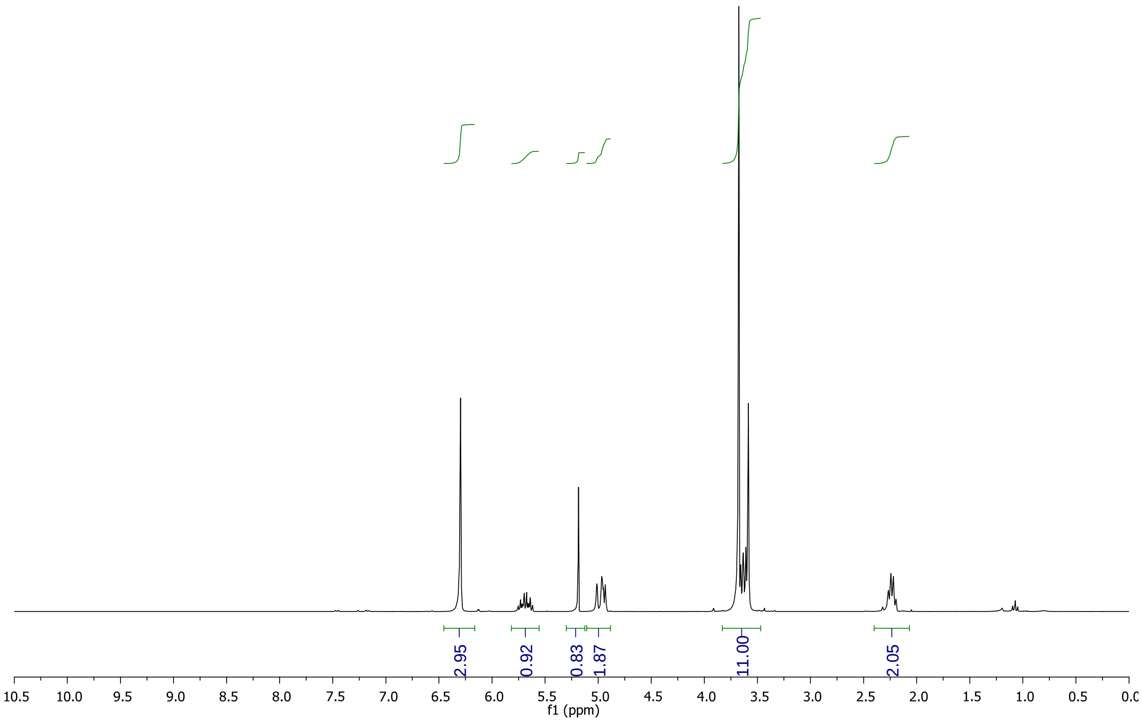

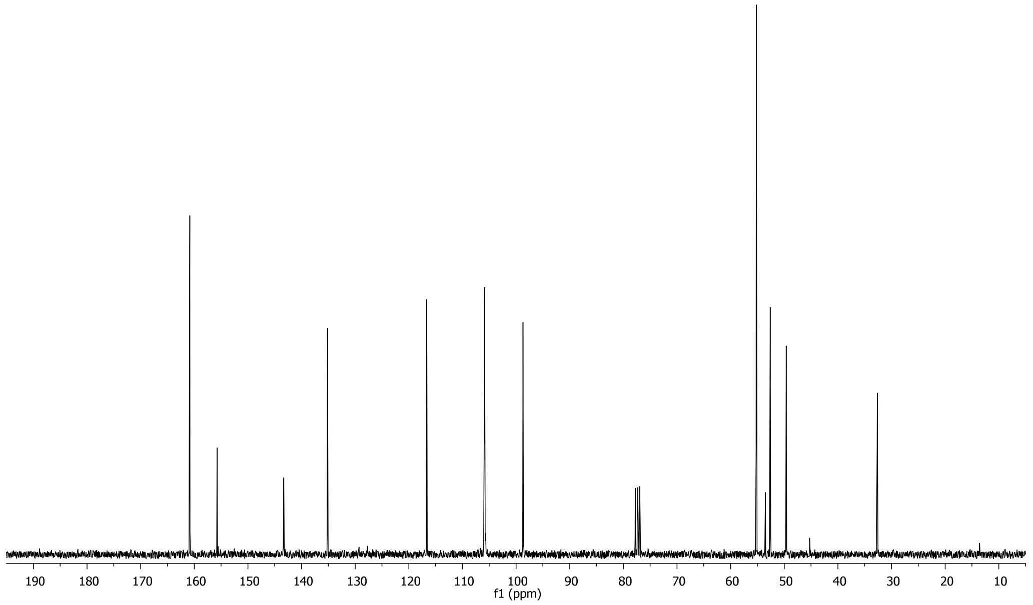

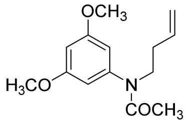
OCH3
H3CO
N
COCH3

1b

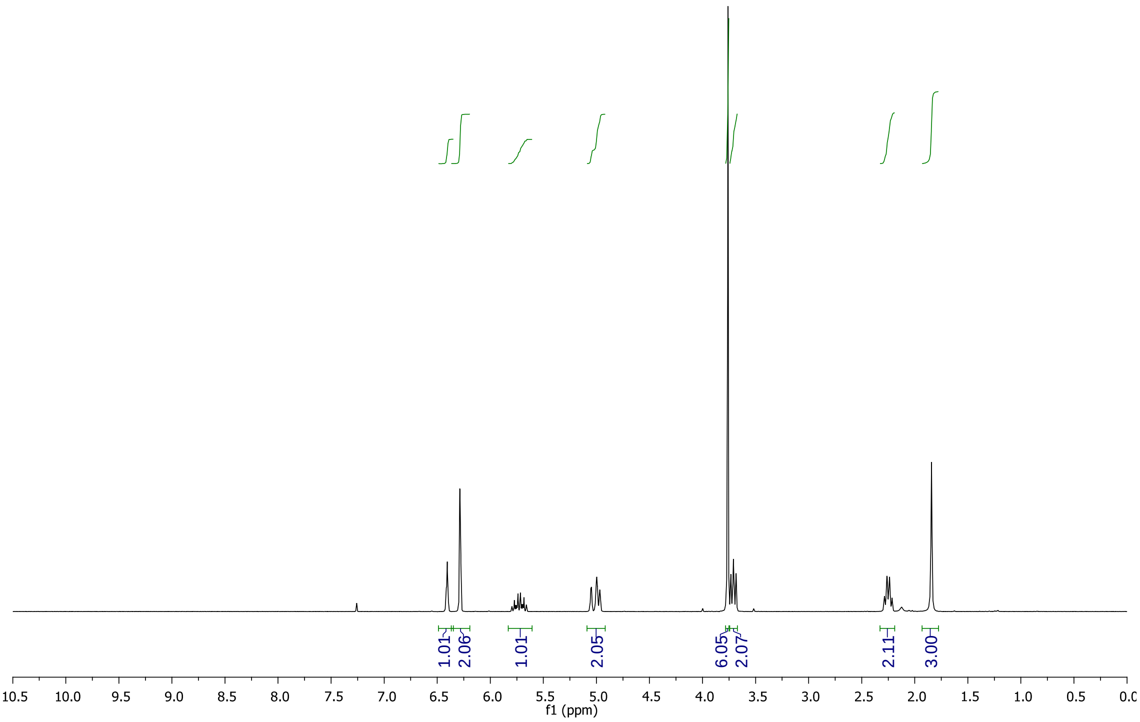
1.01
2.06
1.01
2.05
6.05
2.07
2.11
3.00
10.5 10.0 9.5 9.0 8.5 8.0 7.5 7.0 6.5 6.0 5.5 5.0 4.5 4.0 3.5 3.0 2.5 2.0 1.5 1.0 0.5 0.0
f1 (ppm)

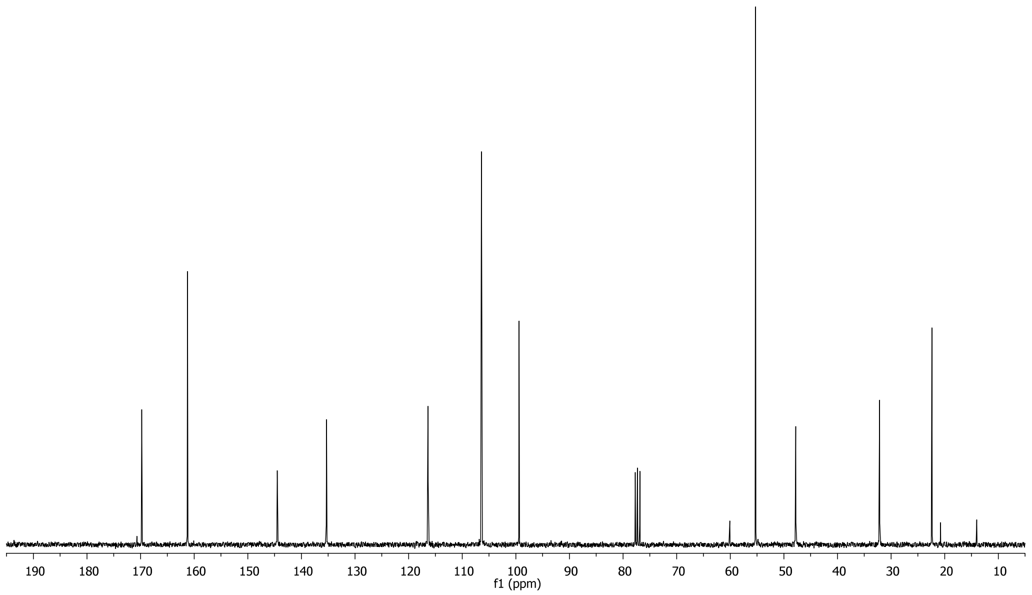
190 180 170 160 150 140 130 120 110 100 90 80 70 60 50 40 30 20 10
f1 (ppm)

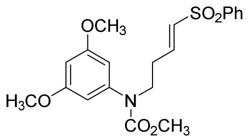
OCH3
SO2Ph
H3CO
N
CO2CH3

1c

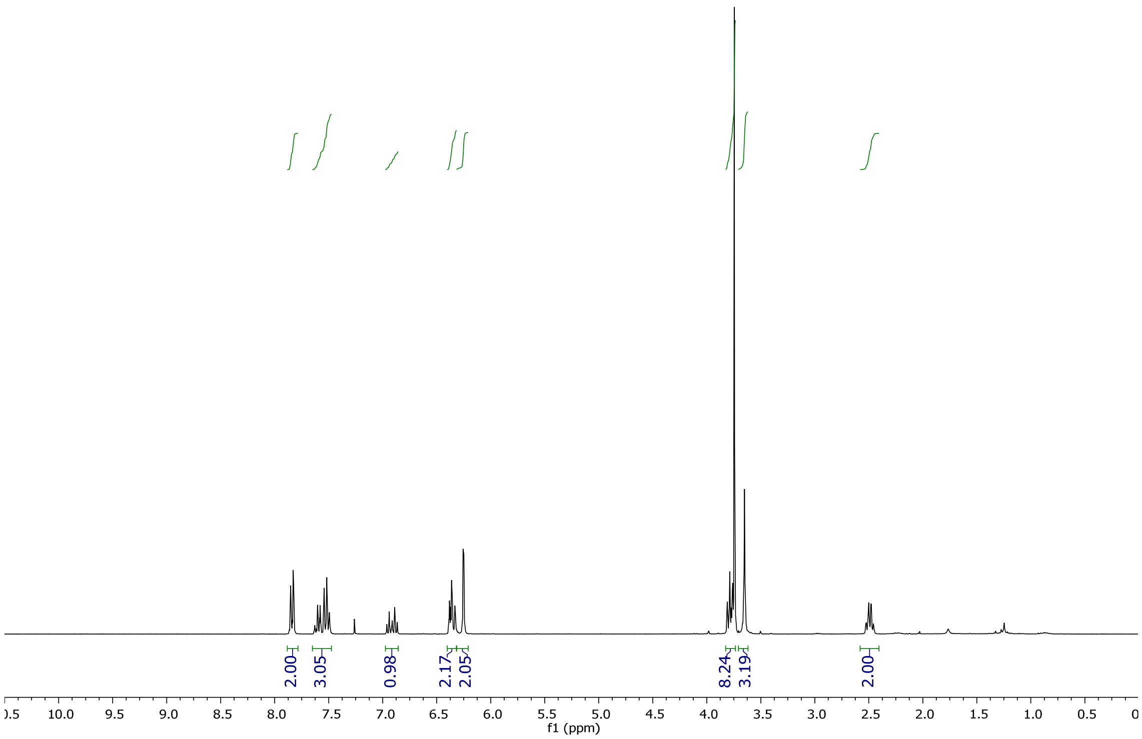
2.00
3.05
0.98
2.17
2.05
8.24
3.19
2.00
f1 (ppm)

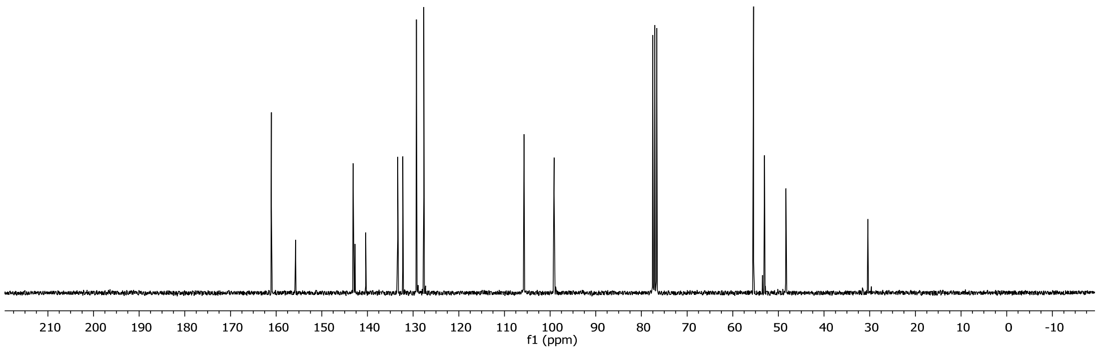
f1 (ppm)

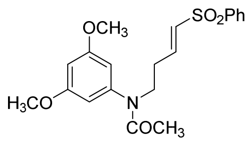

OCH3
SO2Ph
H3CO
N
COCH3

1d

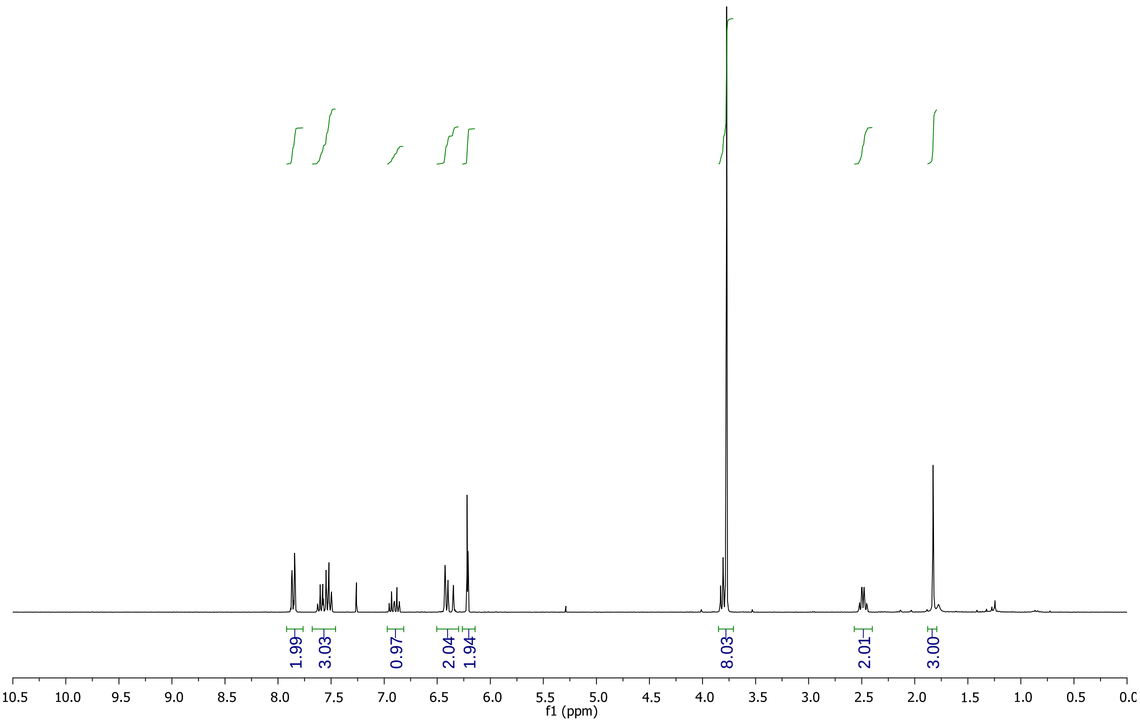

1.99
3.03
0.97
2.04
1.94
8.03
2.01
3.00
10.5 10.0 9.5 9.0 8.5 8.0 7.5 7.0 6.5 6.0 5.5 5.0 4.5 4.0 3.5 3.0 2.5 2.0 1.5 1.0 0.5 0.0
f1 (ppm)

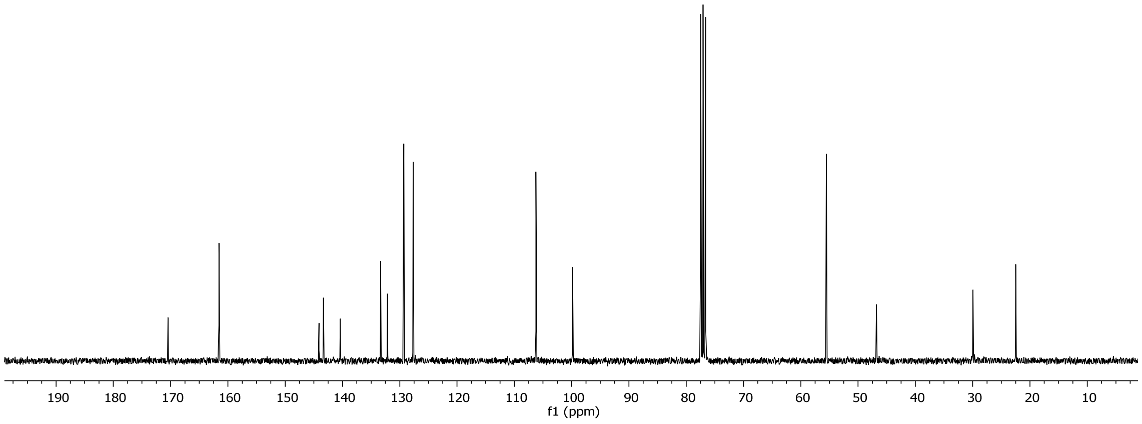

190 180 170 160 150 140 130 120 110 100 90 80 70 60 50 40 30 20 10
f1 (ppm)

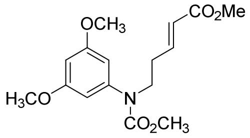

1e

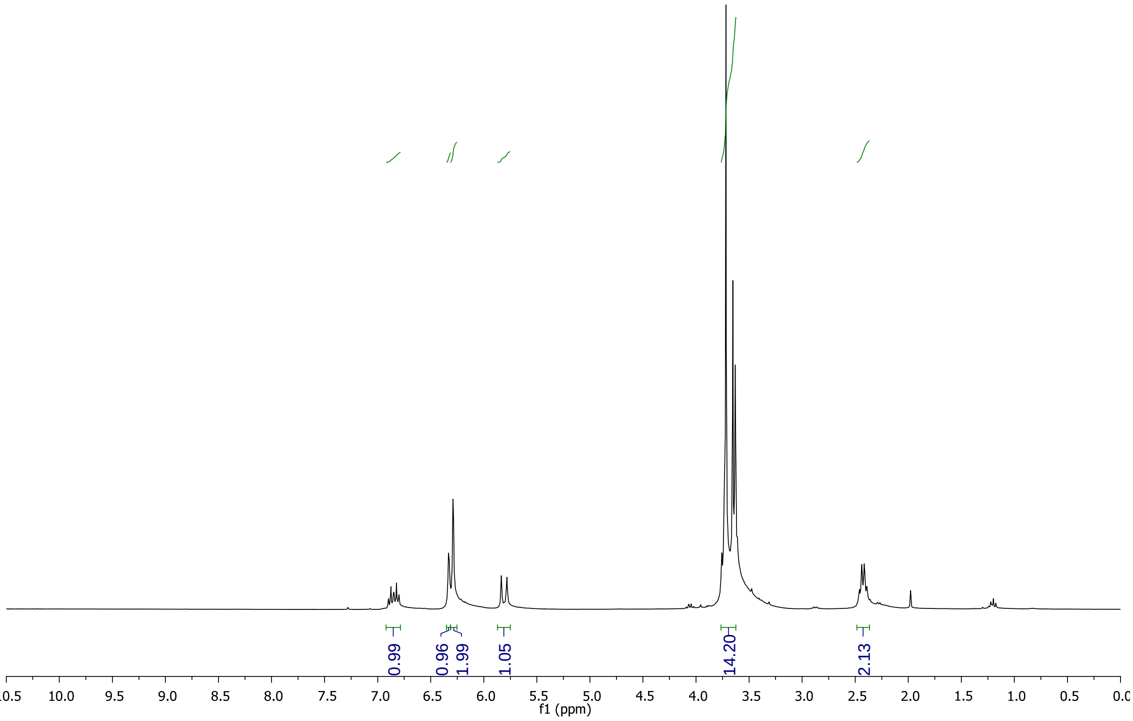

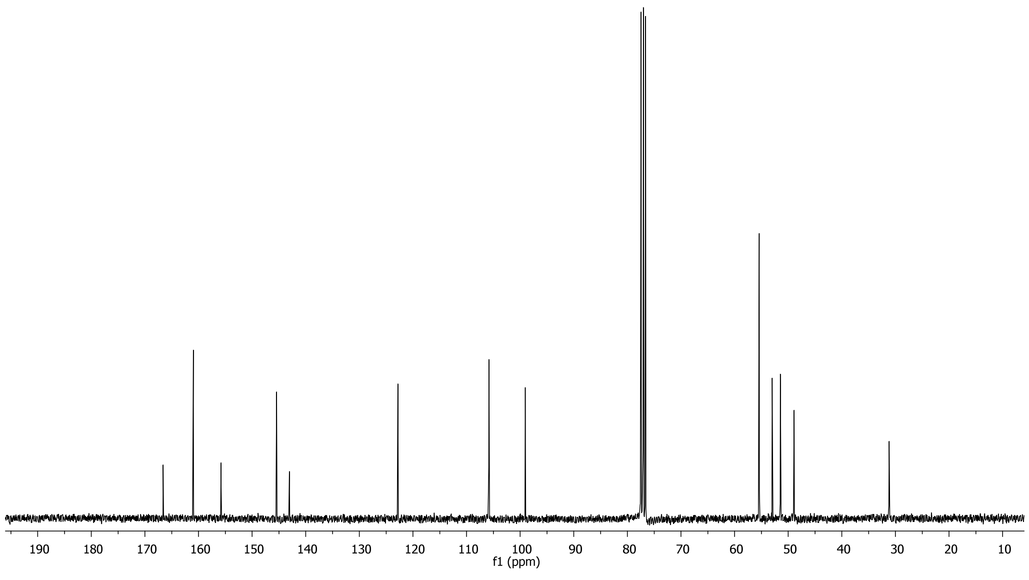

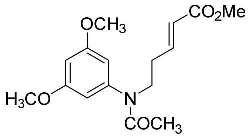
OCH3
CO2Me
H3CO
N
COCH3

1f

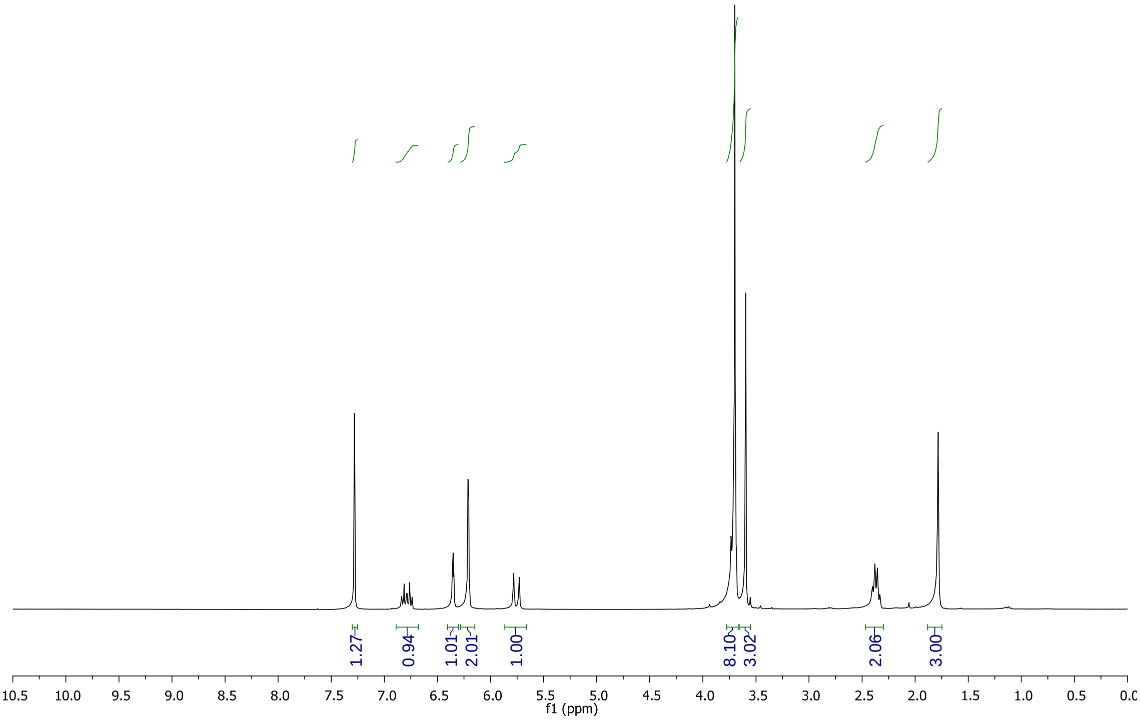
1.27
0.94
1.01
2.01
1.00
8.10
3.02
2.06
3.00
10.5 10.0 9.5 9.0 8.5 8.0 7.5 7.0 6.5 6.0 5.5 5.0 4.5 4.0 3.5 3.0 2.5 2.0 1.5 1.0 0.5
f1 (ppm)

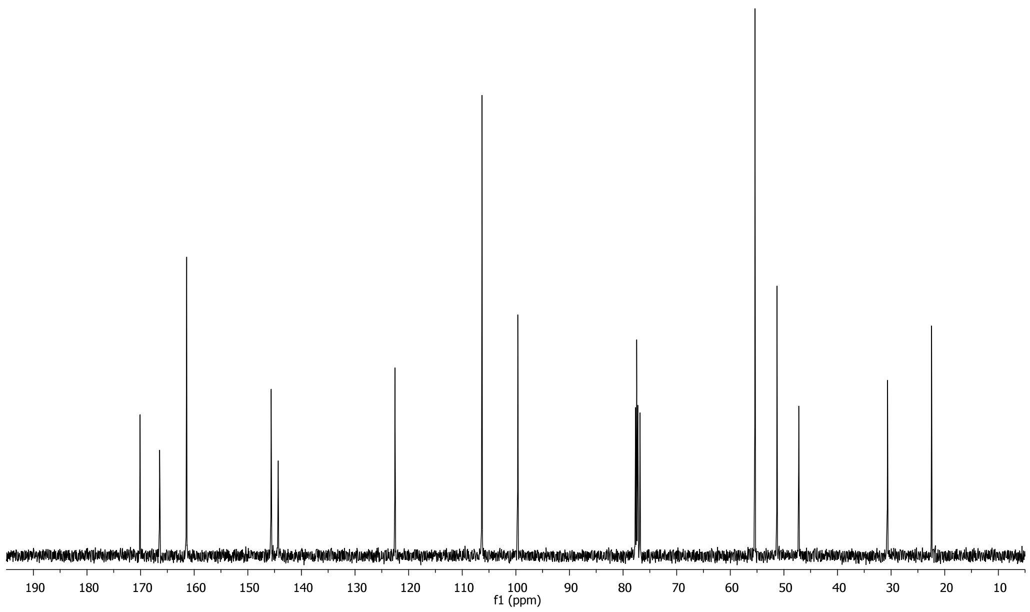
190 180 170 160 150 140 130 120 110 100 90 80 70 60 50 40 30 20 10
f1 (ppm)

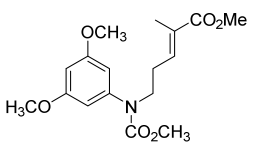
OCH3
CO2Me
H3CO
N
CO2CH3

1g

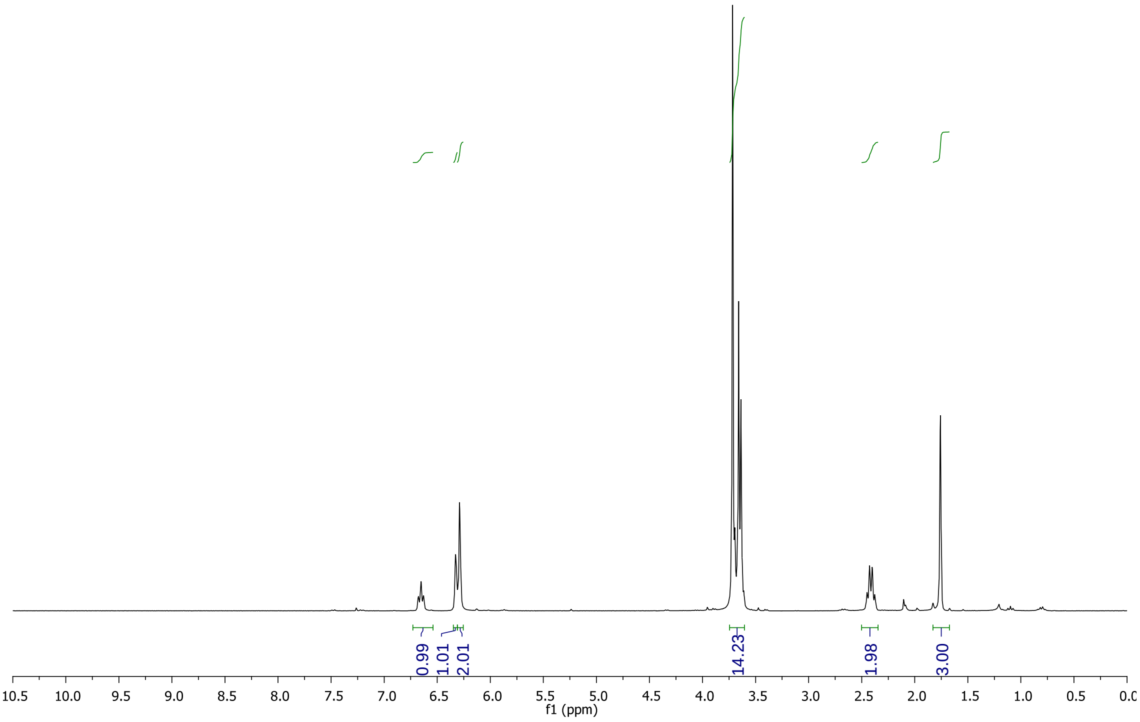
0.99
1.01
2.01
14.23
1.98
3.00
10.5 10.0 9.5 9.0 8.5 8.0 7.5 7.0 6.5 6.0 5.5 5.0 4.5 4.0 3.5 3.0 2.5 2.0 1.5 1.0 0.5 0.0
f1 (ppm)

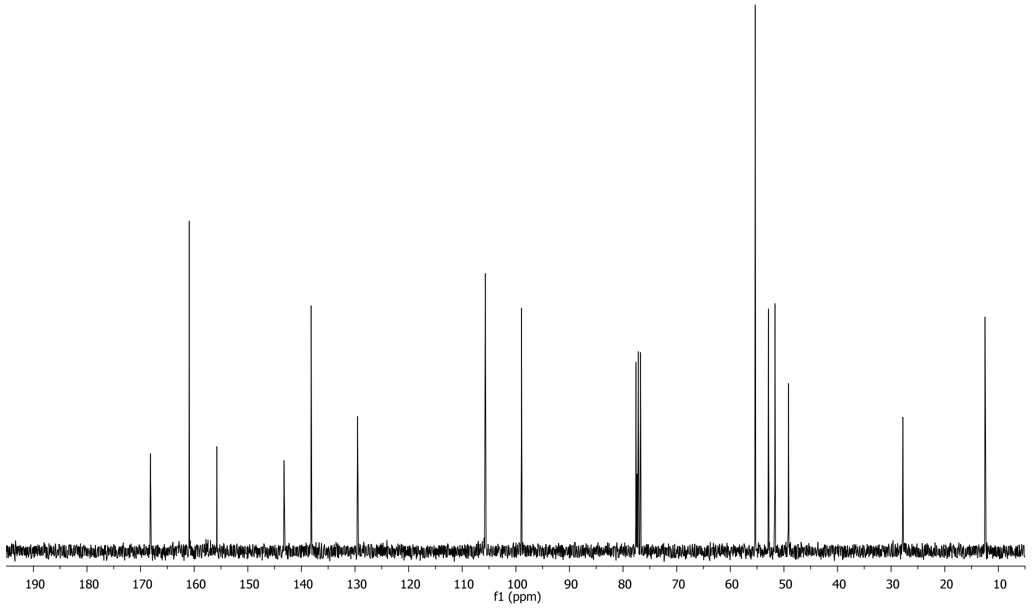
190 180 170 160 150 140 130 120 110 100 90 80 70 60 50 40 30 20 10
f1 (ppm)

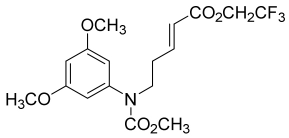
OCH3
CO2CH2CF3
H3CO
N
CO2CH3

1h

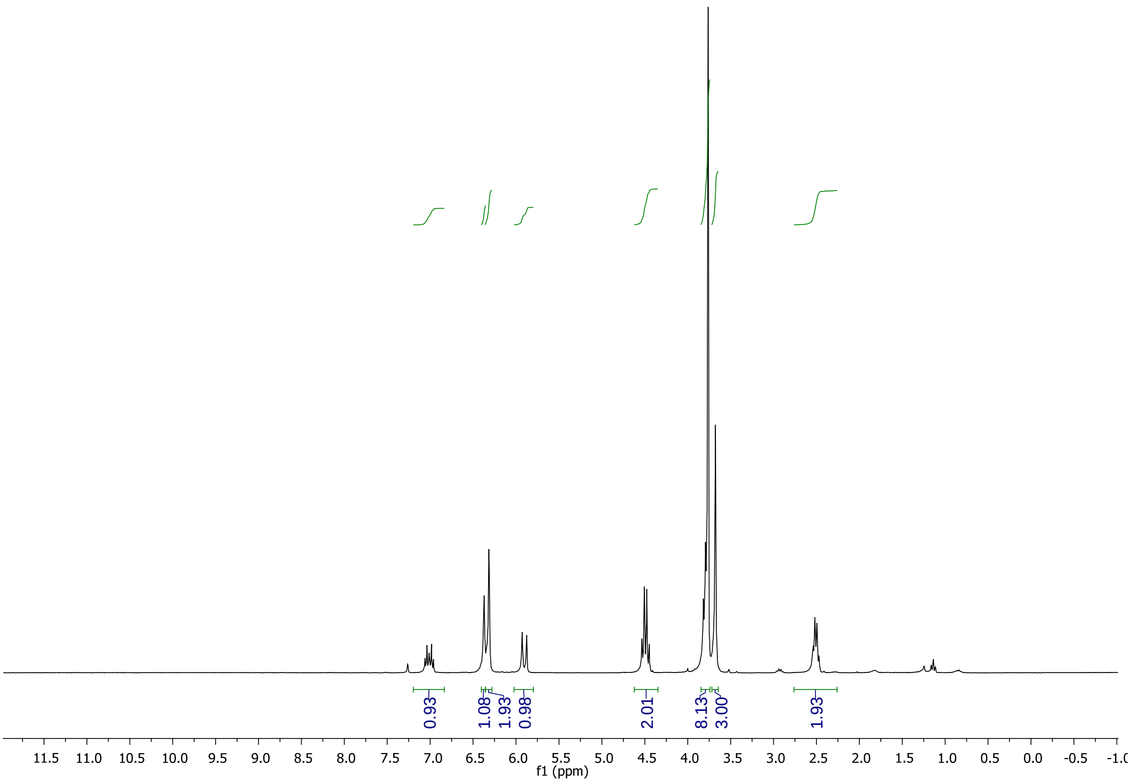
0.93
1.08
1.93
0.98
2.01
8.13
3.00
1.93
f1 (ppm)

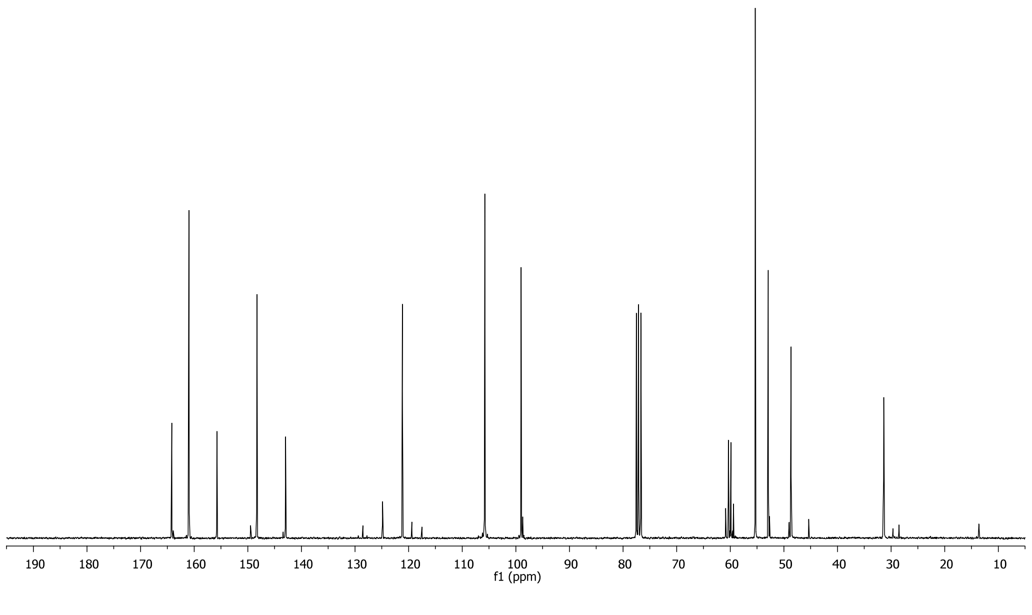
f1 (ppm)

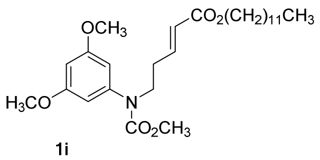
OCH$_3$
CO$_2$(CH$_2$)$_{11}$CH$_3$
H$_3$CO
N
CO$_2$CH$_3$

1i

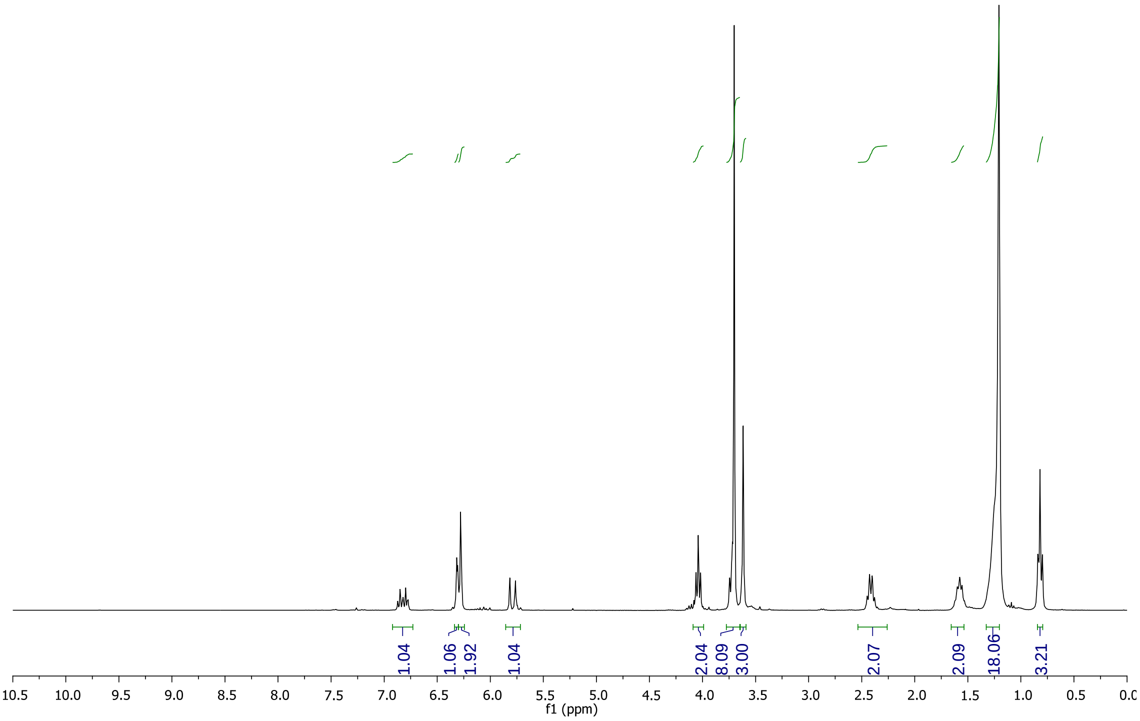
1.04
1.06
1.92
1.04
2.04
8.09
3.00
2.07
2.09
18.06
3.21
10.5 10.0 9.5 9.0 8.5 8.0 7.5 7.0 6.5 6.0 5.5 5.0 4.5 4.0 3.5 3.0 2.5 2.0 1.5 1.0 0.5 0.0
f1 (ppm)

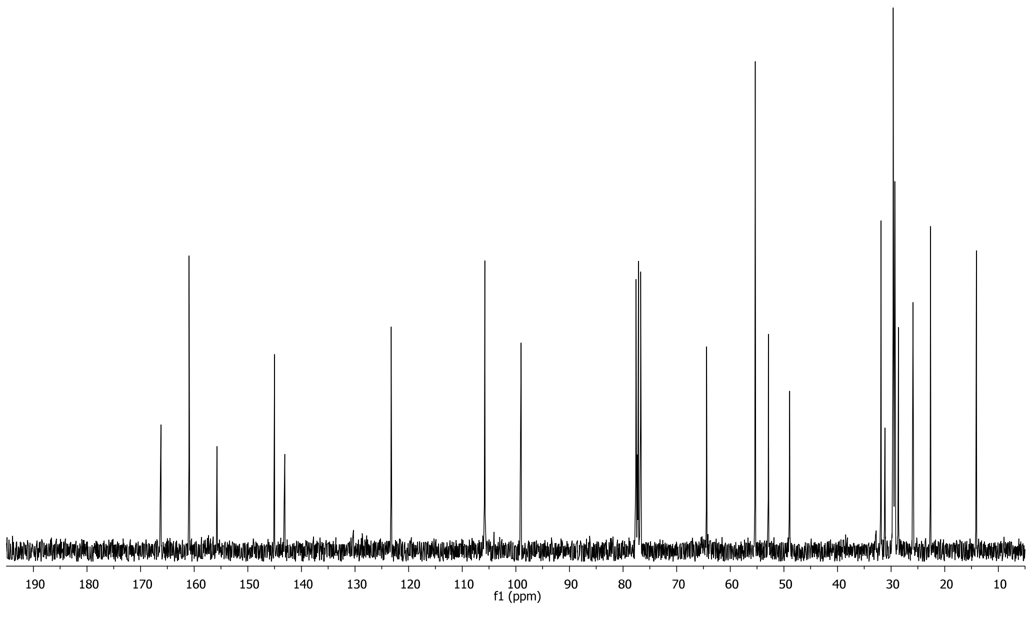
190 180 170 160 150 140 130 120 110 100 90 80 70 60 50 40 30 20 10
f1 (ppm)

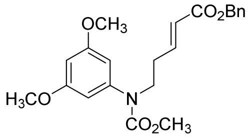
OCH3
CO2Bn
H3CO
N
CO2CH3

1j

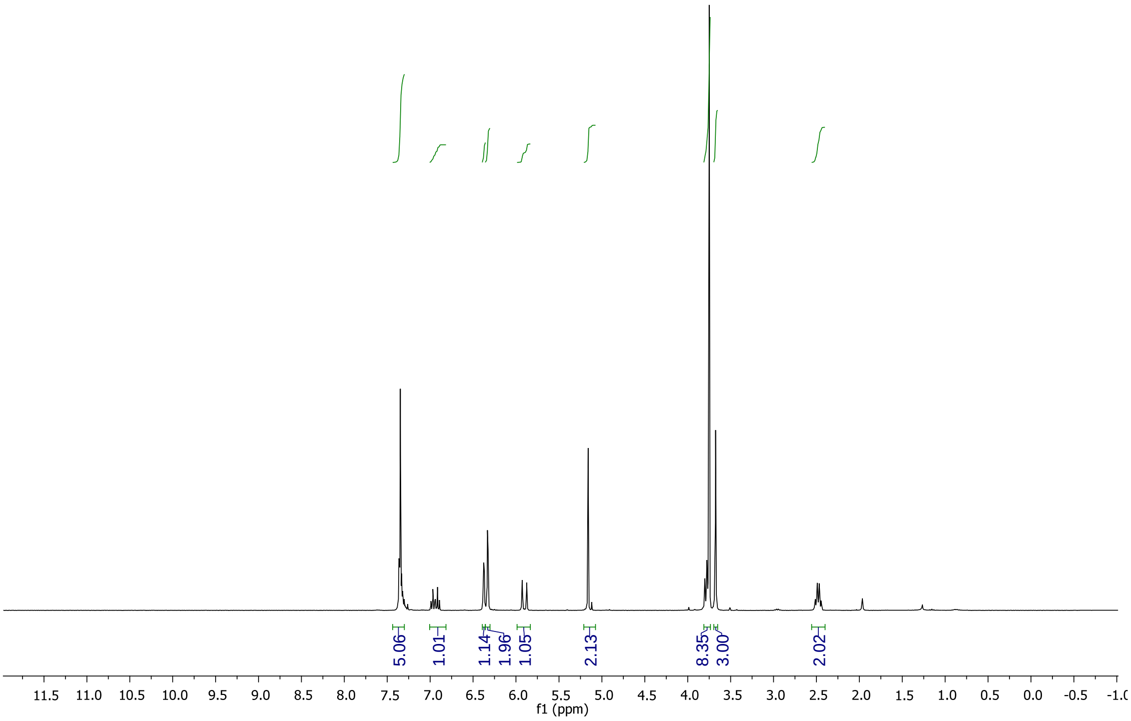
5.06
1.01
1.14
1.96
1.05
2.13
8.35
3.00
2.02
11.5 11.0 10.5 10.0 9.5 9.0 8.5 8.0 7.5 7.0 6.5 6.0 5.5 5.0 4.5 4.0 3.5 3.0 2.5 2.0 1.5 1.0 0.5 0.0 -0.5 -1.0
f1 (ppm)

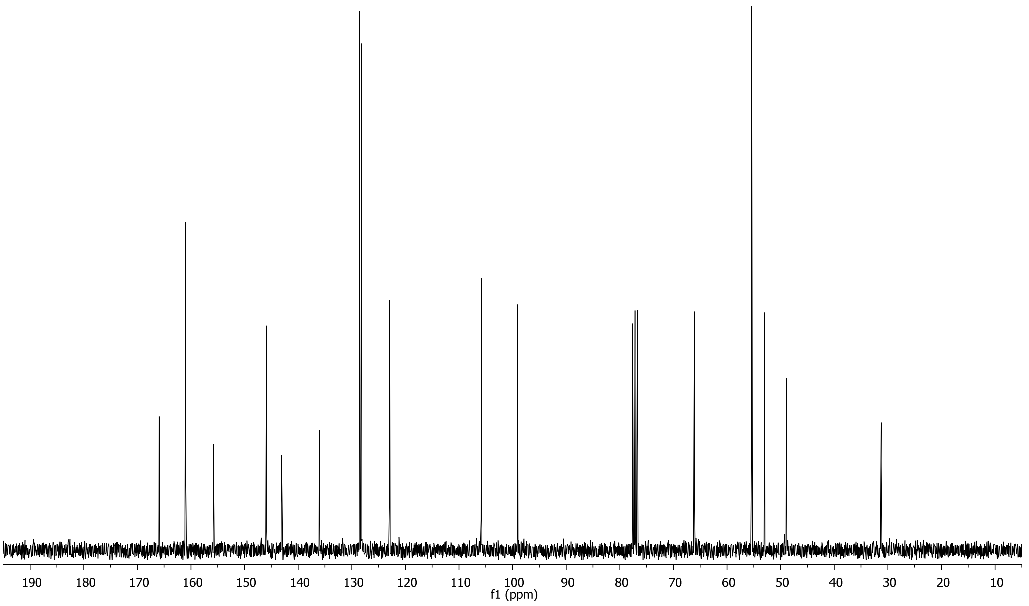
190 180 170 160 150 140 130 120 110 100 90 80 70 60 50 40 30 20 10
f1 (ppm)

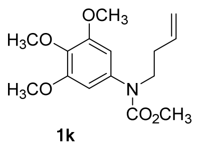
OCH3
H3CO
H3CO
N
CO2CH3

1k

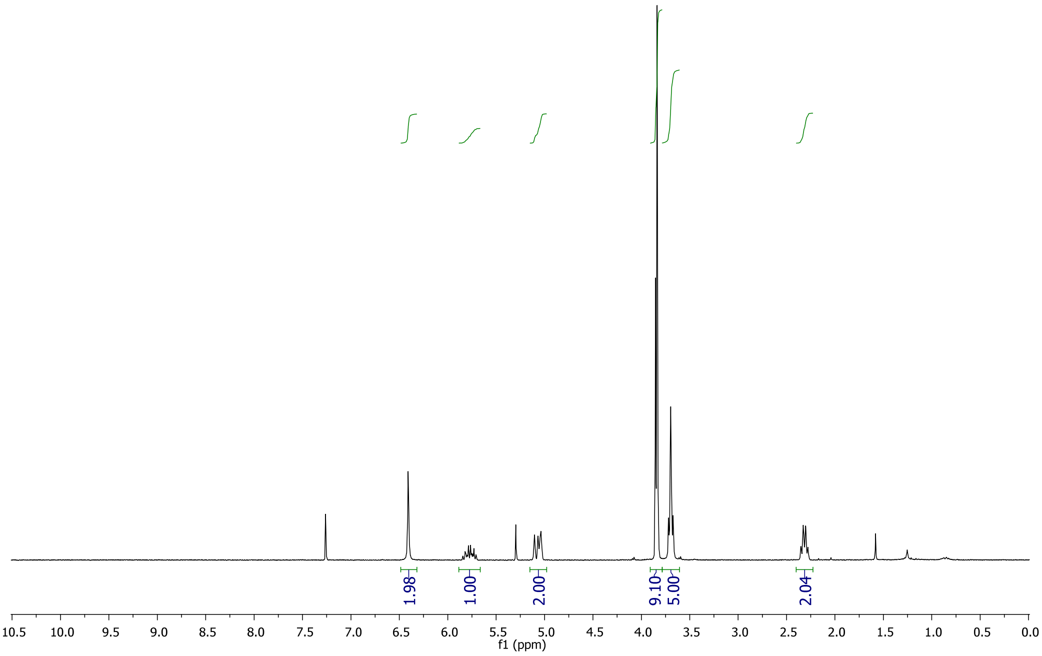
1.98
1.00
2.00
9.10
5.00
2.04
10.5 10.0 9.5 9.0 8.5 8.0 7.5 7.0 6.5 6.0 5.5 5.0 4.5 4.0 3.5 3.0 2.5 2.0 1.5 1.0 0.5 0.0
f1 (ppm)

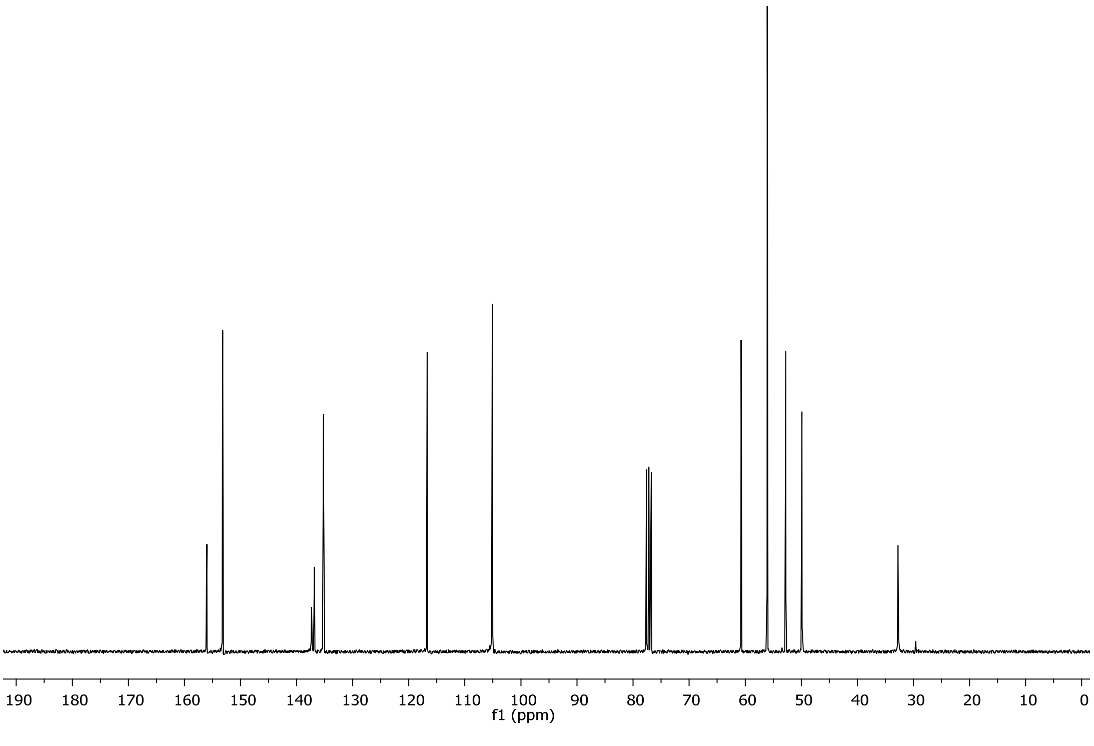
190 180 170 160 150 140 130 120 110 100 90 80 70 60 50 40 30 20 10 0
f1 (ppm)

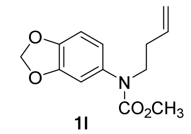
O
O
N
CO2CH3

1l

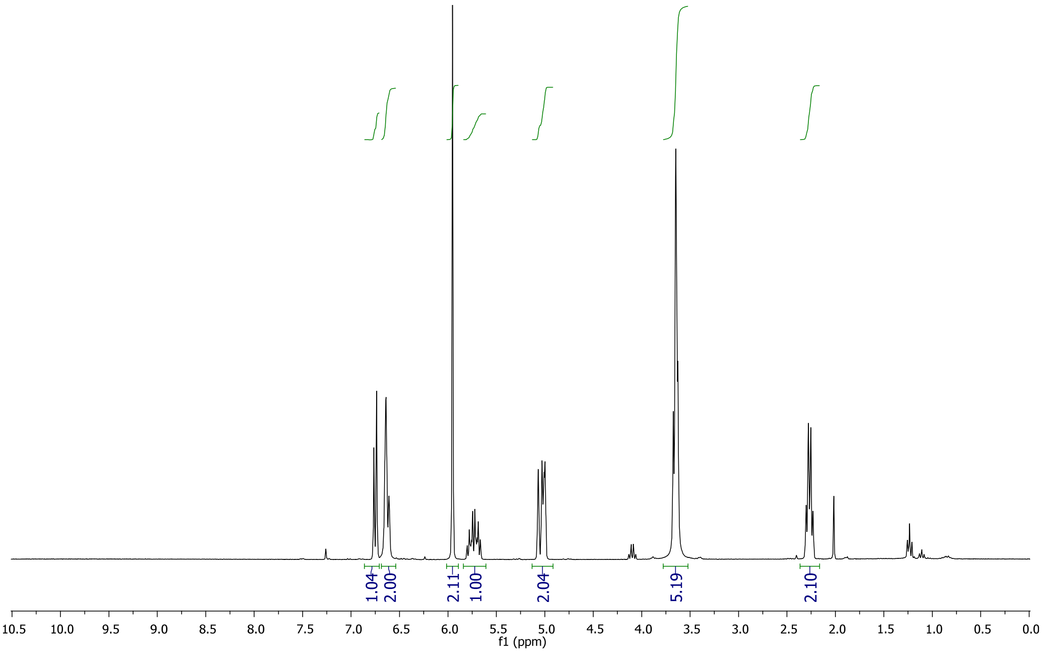
1.04
2.00
2.11
1.00
2.04
5.19
2.10
10.5 10.0 9.5 9.0 8.5 8.0 7.5 7.0 6.5 6.0 5.5 5.0 4.5 4.0 3.5 3.0 2.5 2.0 1.5 1.0 0.5 0.0
f1 (ppm)

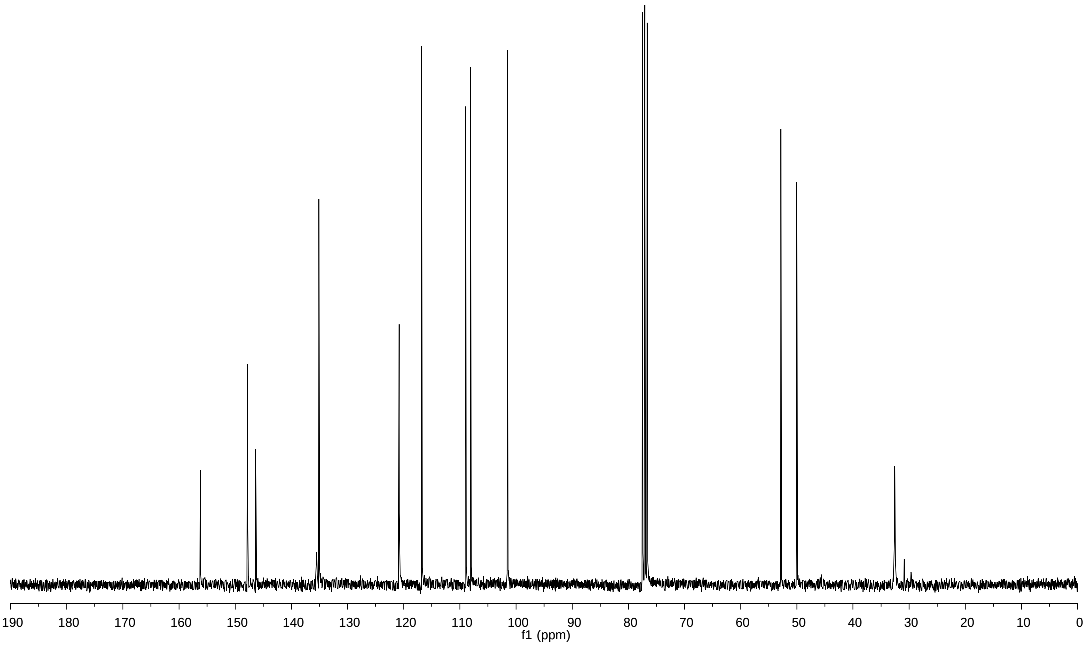
190 180 170 160 150 140 130 120 110 100 90 80 70 60 50 40 30 20 10 0
f1 (ppm)

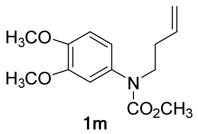
H3CO
H3CO
N
CO2CH3

1m

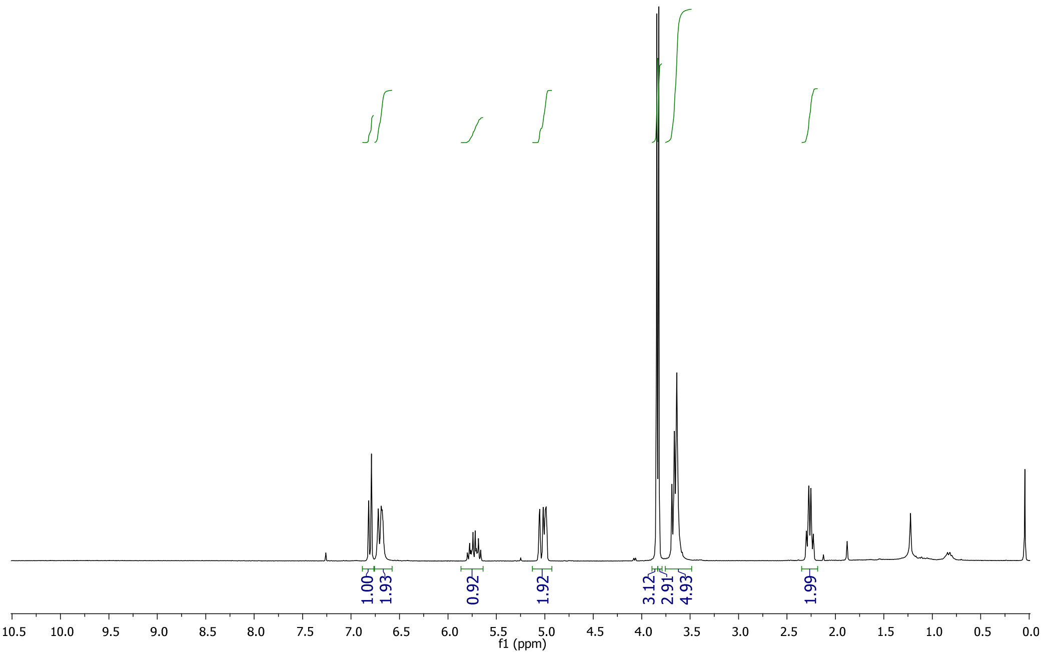
1.00
1.93
0.92
1.92
3.12
2.91
4.93
1.99
10.5 10.0 9.5 9.0 8.5 8.0 7.5 7.0 6.5 6.0 5.5 5.0 4.5 4.0 3.5 3.0 2.5 2.0 1.5 1.0 0.5 0.0
f1 (ppm)

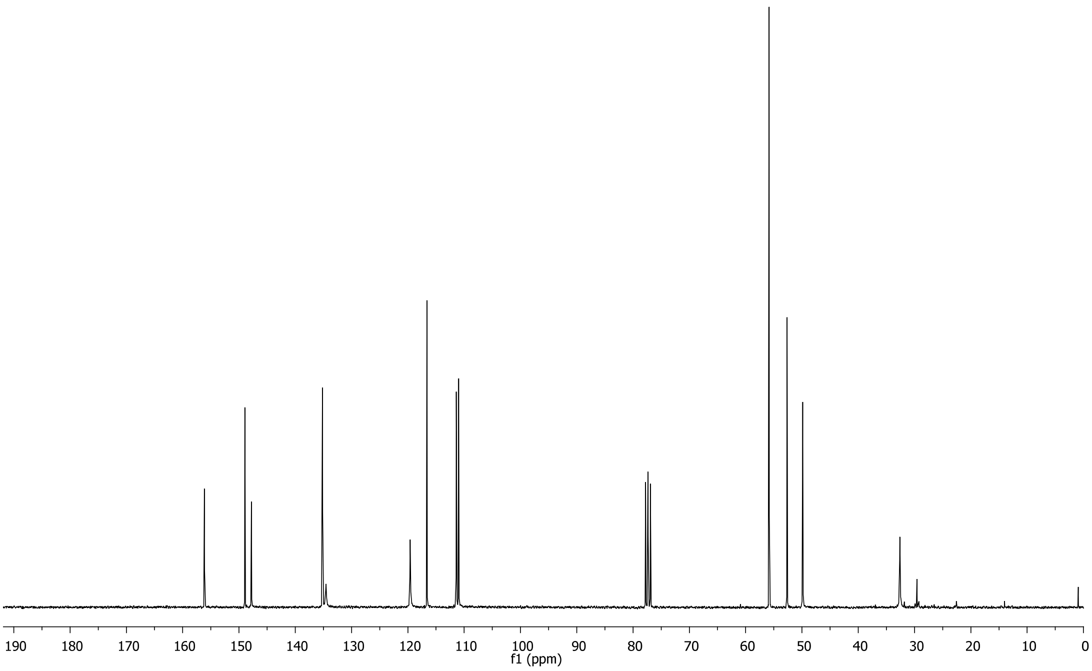
190 180 170 160 150 140 130 120 110 100 90 80 70 60 50 40 30 20 10 0
f1 (ppm)

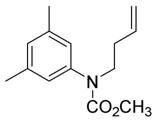

1n

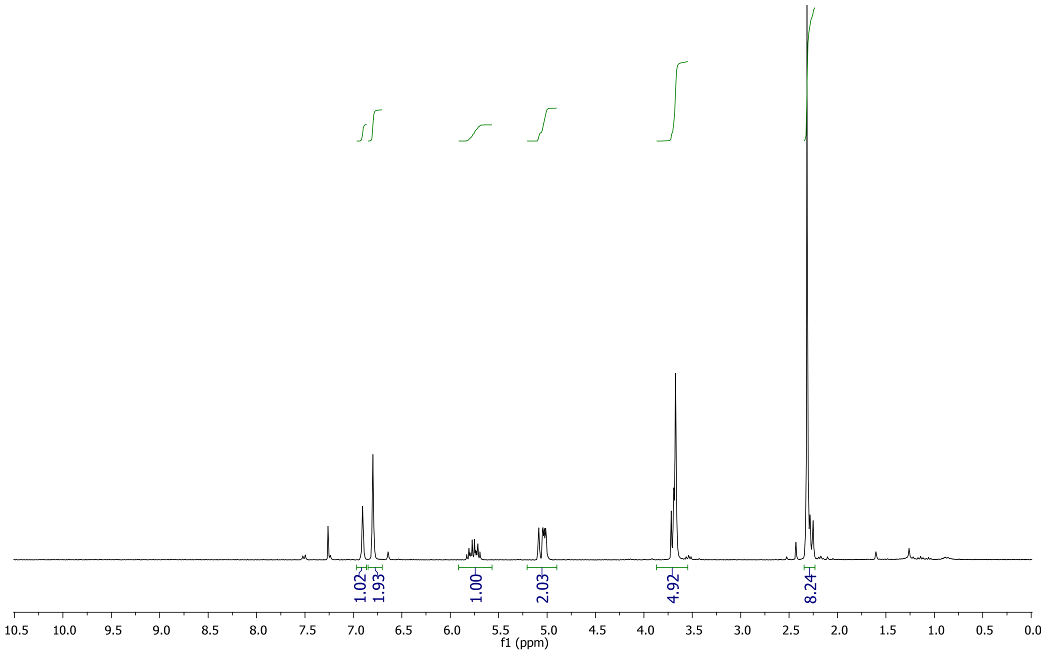

1.02
1.93
1.00
2.03
4.92
8.24
10.5 10.0 9.5 9.0 8.5 8.0 7.5 7.0 6.5 6.0 5.5 5.0 4.5 4.0 3.5 3.0 2.5 2.0 1.5 1.0 0.5 0.0
f1 (ppm)

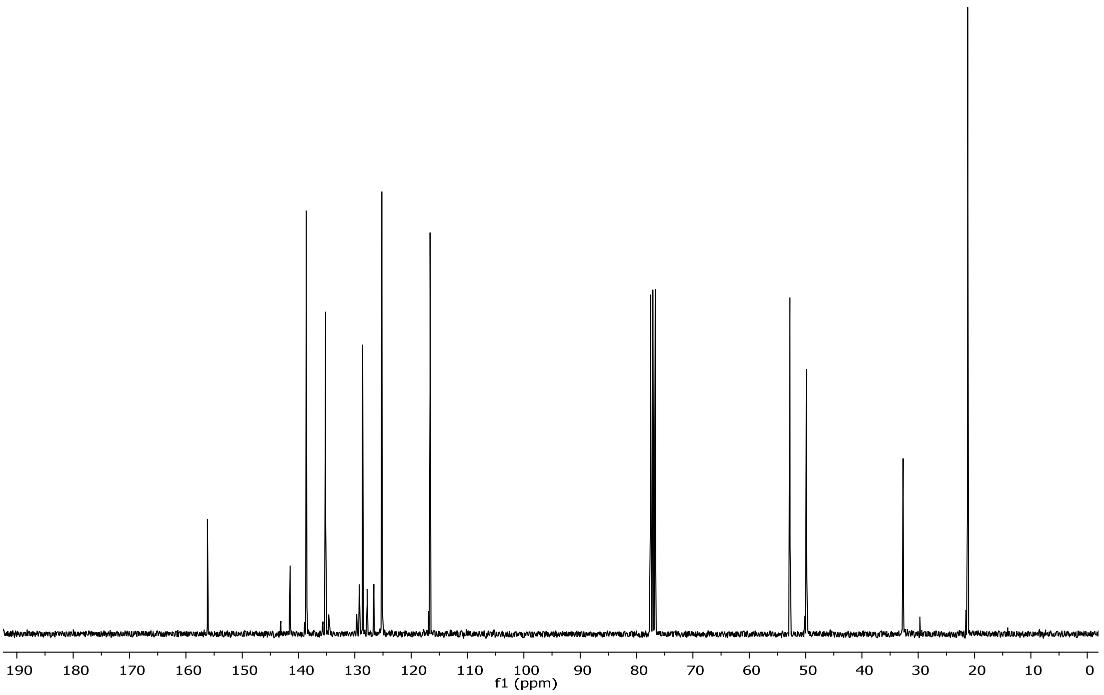

190 180 170 160 150 140 130 120 110 100 90 80 70 60 50 40 30 20 10 0
f1 (ppm)

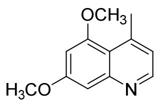
OCH3
H3CO
N

2a

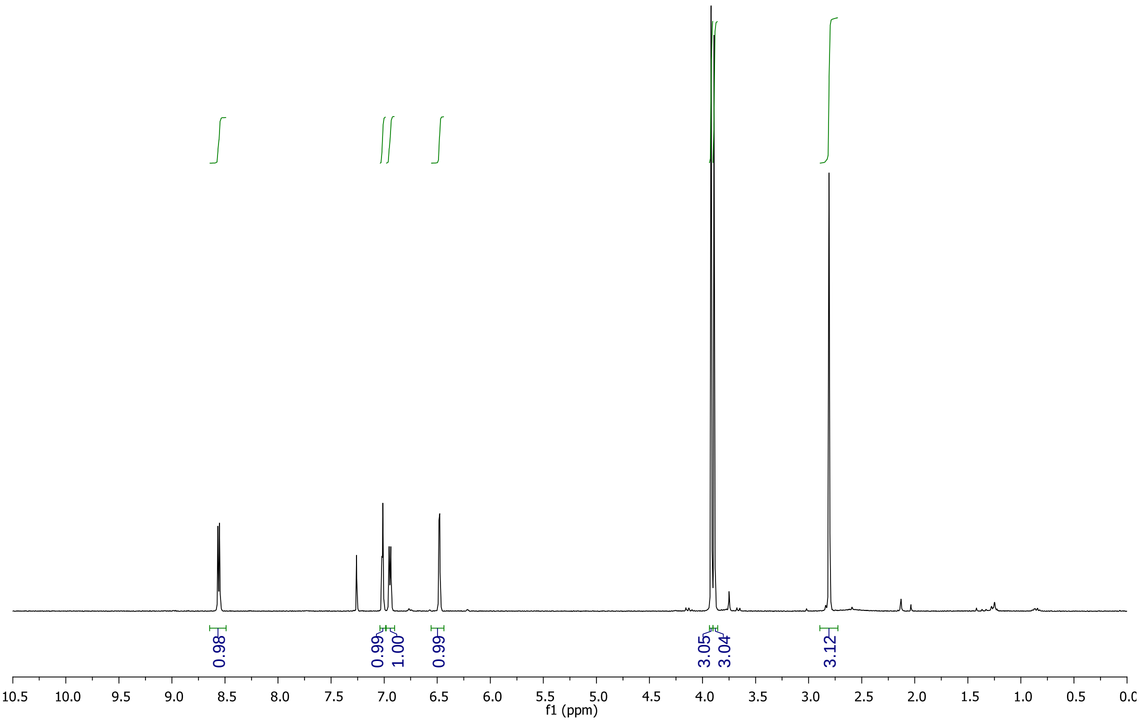
0.98
0.99
1.00
0.99
3.05
3.04
3.12
10.5 10.0 9.5 9.0 8.5 8.0 7.5 7.0 6.5 6.0 5.5 5.0 4.5 4.0 3.5 3.0 2.5 2.0 1.5 1.0 0.5 0.0
f1 (ppm)

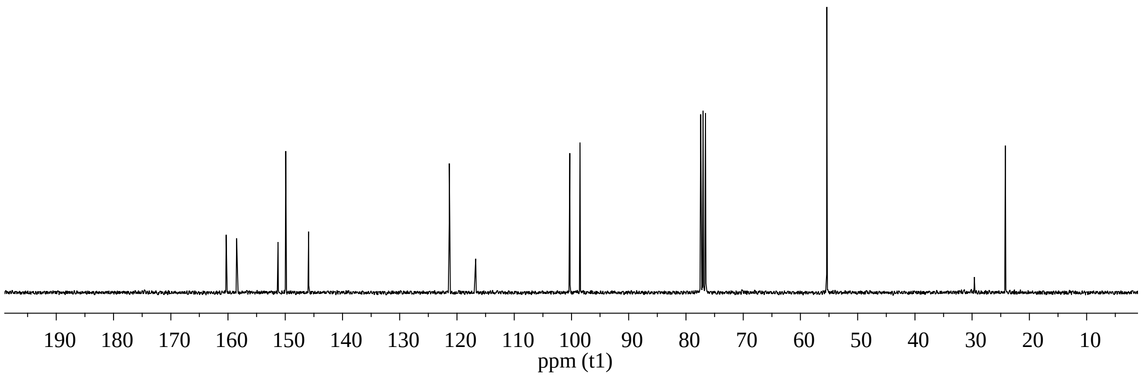
190 180 170 160 150 140 130 120 110 100 90 80 70 60 50 40 30 20 10
ppm (t1)

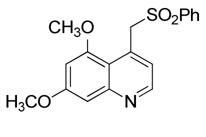
CH3O
SO2Ph
H3CO
N

2b

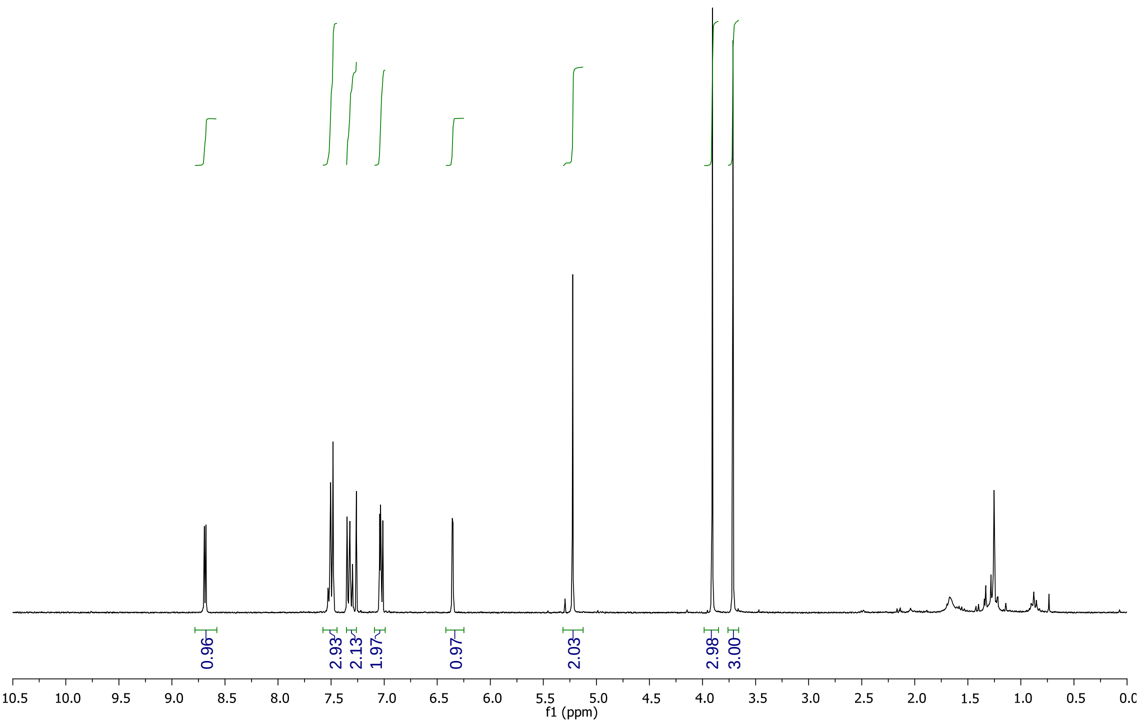
0.96
2.93
2.13
1.97
0.97
2.03
2.98
3.00
10.5 10.0 9.5 9.0 8.5 8.0 7.5 7.0 6.5 6.0 5.5 5.0 4.5 4.0 3.5 3.0 2.5 2.0 1.5 1.0 0.5
f1 (ppm)

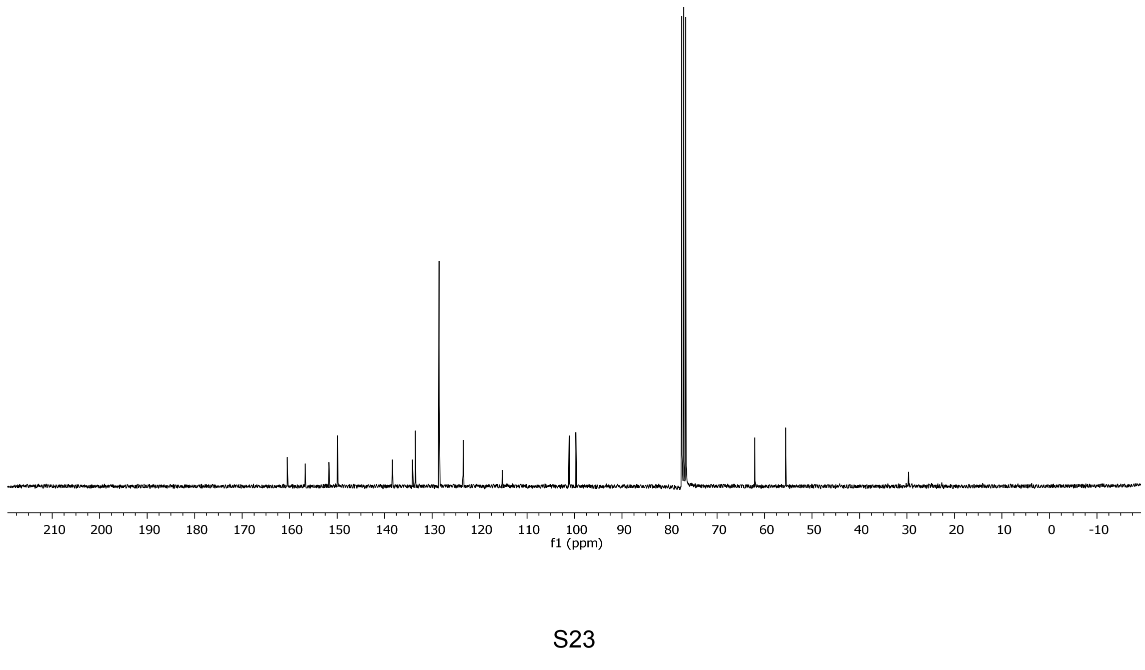
210 200 190 180 170 160 150 140 130 120 110 100 90 80 70 60 50 40 30 20 10 0 -10
f1 (ppm)

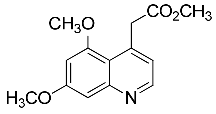
CO2CH3
CH3O
H3CO
N

2c

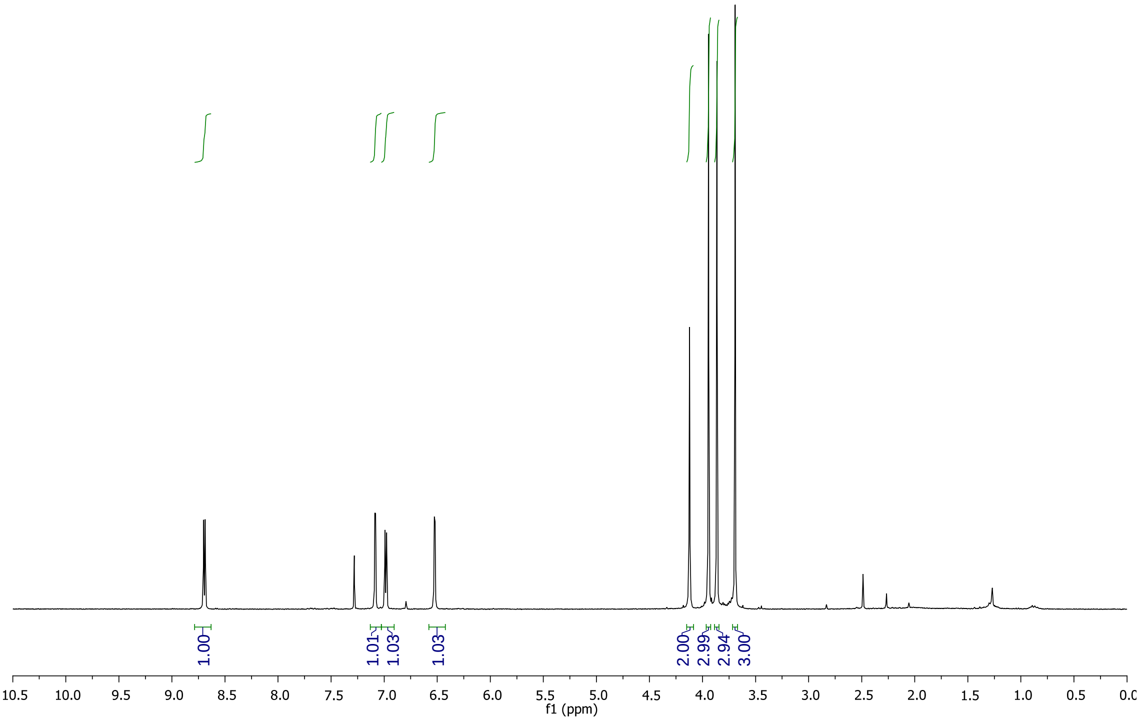
1.00
1.01
1.03
1.03
2.00
2.99
2.94
3.00
10.5 10.0 9.5 9.0 8.5 8.0 7.5 7.0 6.5 6.0 5.5 5.0 4.5 4.0 3.5 3.0 2.5 2.0 1.5 1.0 0.5
f1 (ppm)

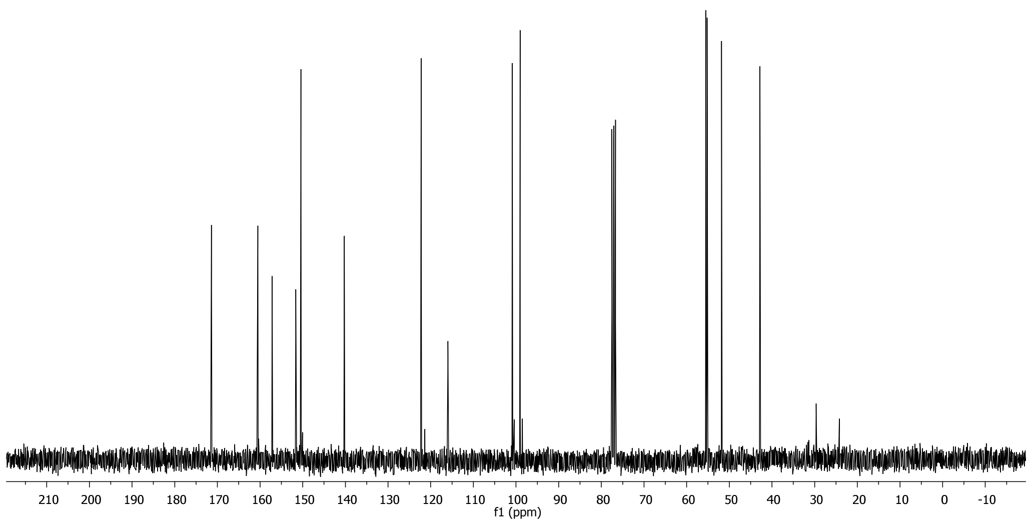
210 200 190 180 170 160 150 140 130 120 110 100 90 80 70 60 50 40 30 20 10 0 -10
f1 (ppm)

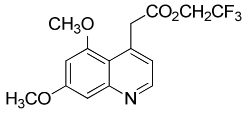
CH3O
CO2CH2CF3
H3CO
N

2d

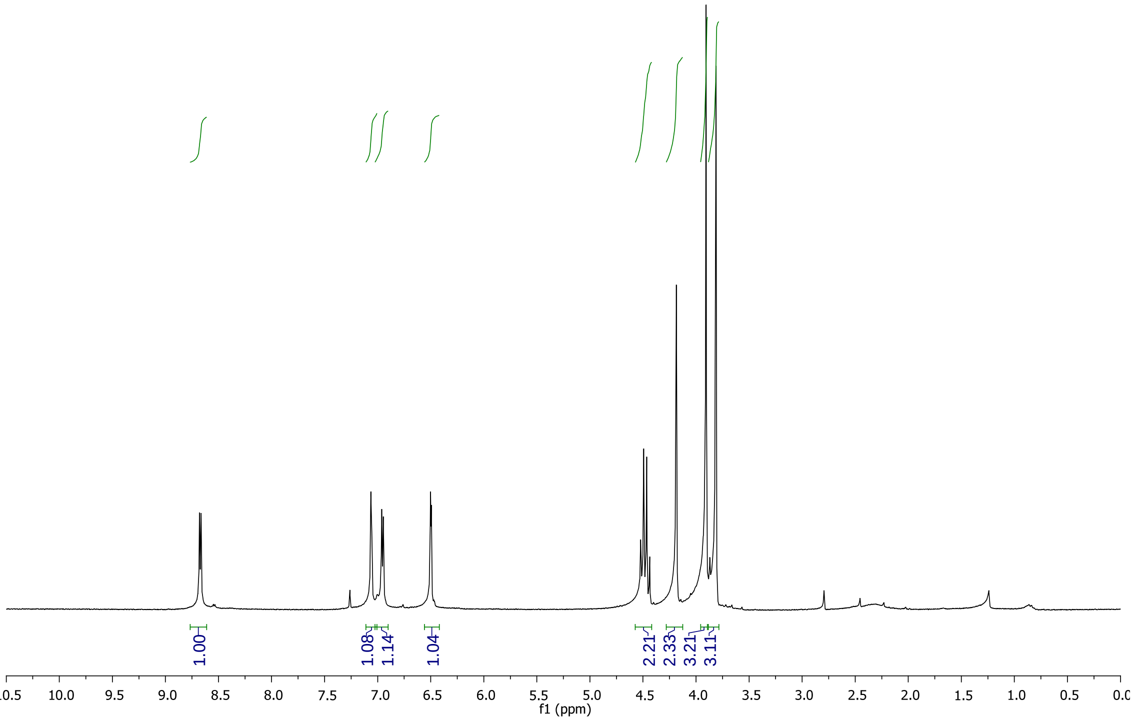
1.00
1.08
1.14
1.04
2.21
2.33
3.21
3.11
10.5 10.0 9.5 9.0 8.5 8.0 7.5 7.0 6.5 6.0 5.5 5.0 4.5 4.0 3.5 3.0 2.5 2.0 1.5 1.0 0.5 0.0
f1 (ppm)

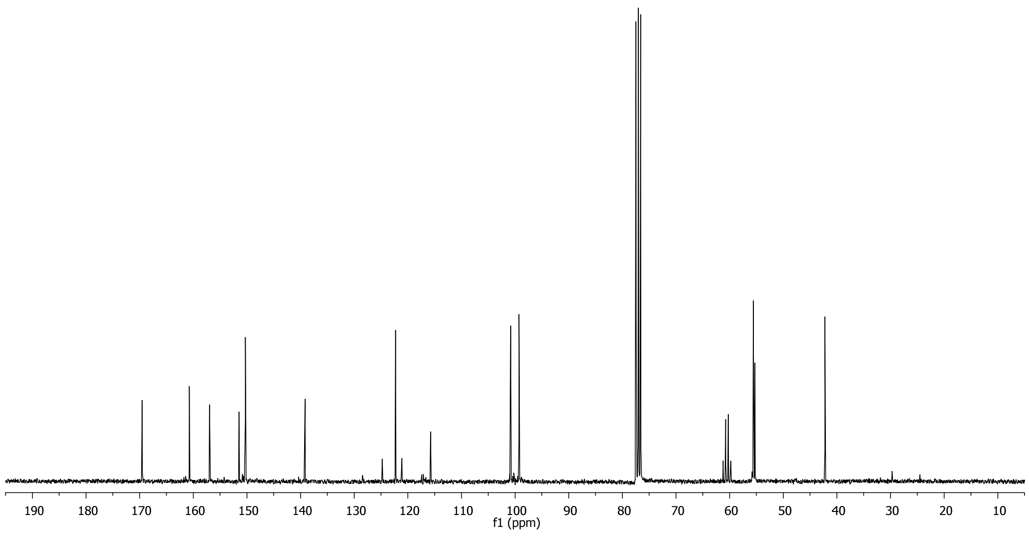
190 180 170 160 150 140 130 120 110 100 90 80 70 60 50 40 30 20 10
f1 (ppm)

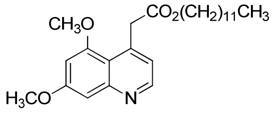
CH3O
CO2(CH2)11CH3
H3CO
N

2e

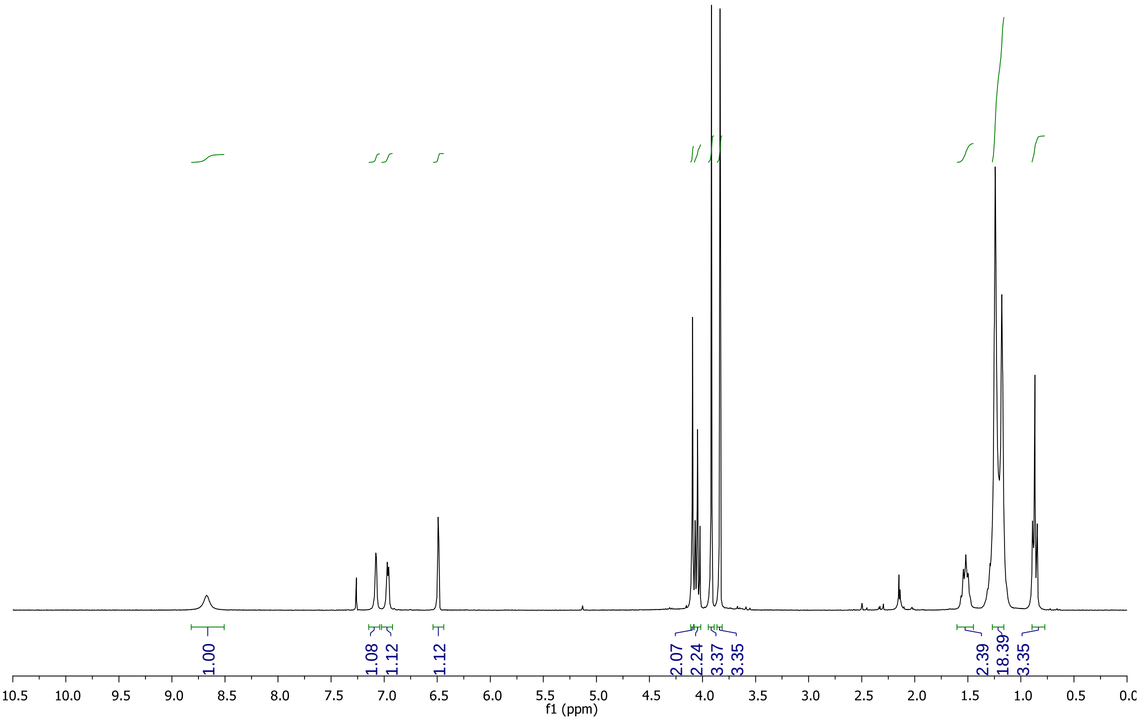
1.00
1.08
1.12
1.12
2.07
2.24
3.37
3.35
2.39
18.39
3.35
10.5 10.0 9.5 9.0 8.5 8.0 7.5 7.0 6.5 6.0 5.5 5.0 4.5 4.0 3.5 3.0 2.5 2.0 1.5 1.0 0.5 0.0
f1 (ppm)

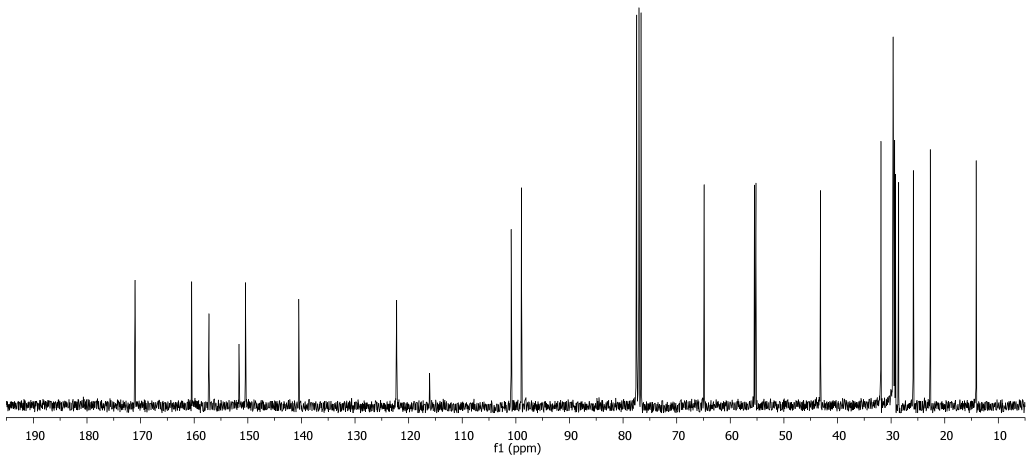
190 180 170 160 150 140 130 120 110 100 90 80 70 60 50 40 30 20 10
f1 (ppm)

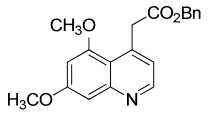
CH3O
CO2Bn
H3CO
N

2f

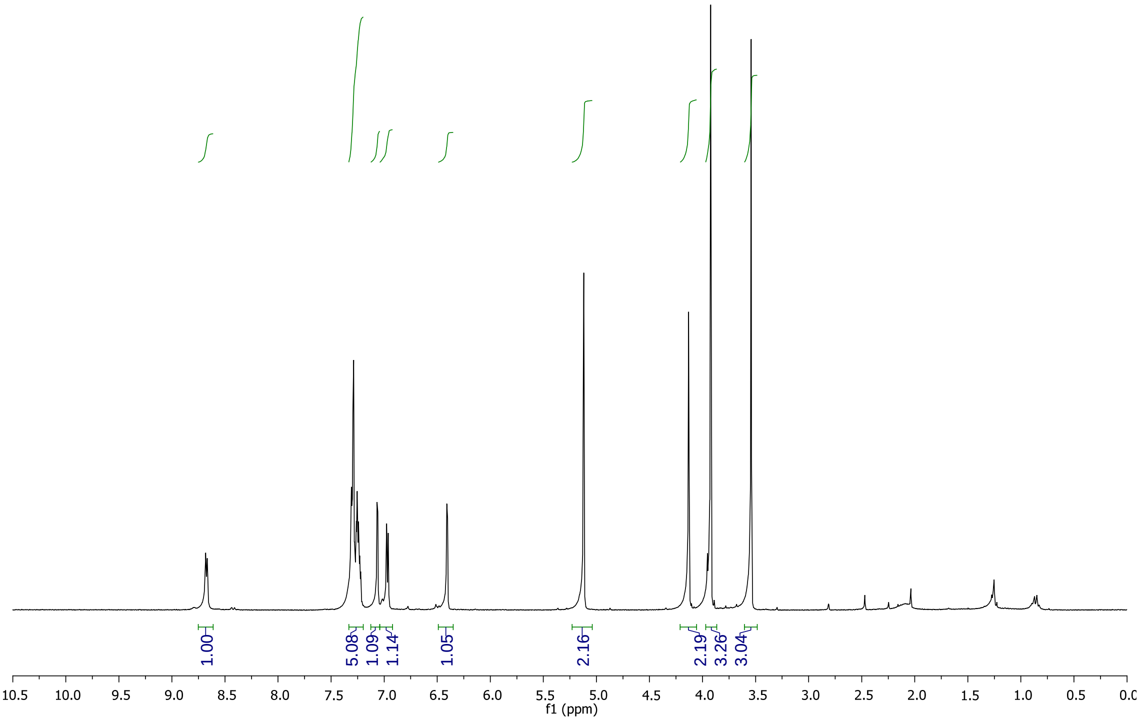
1.00
5.08
1.09
1.14
1.05
2.16
2.19
3.26
3.04
f1 (ppm)

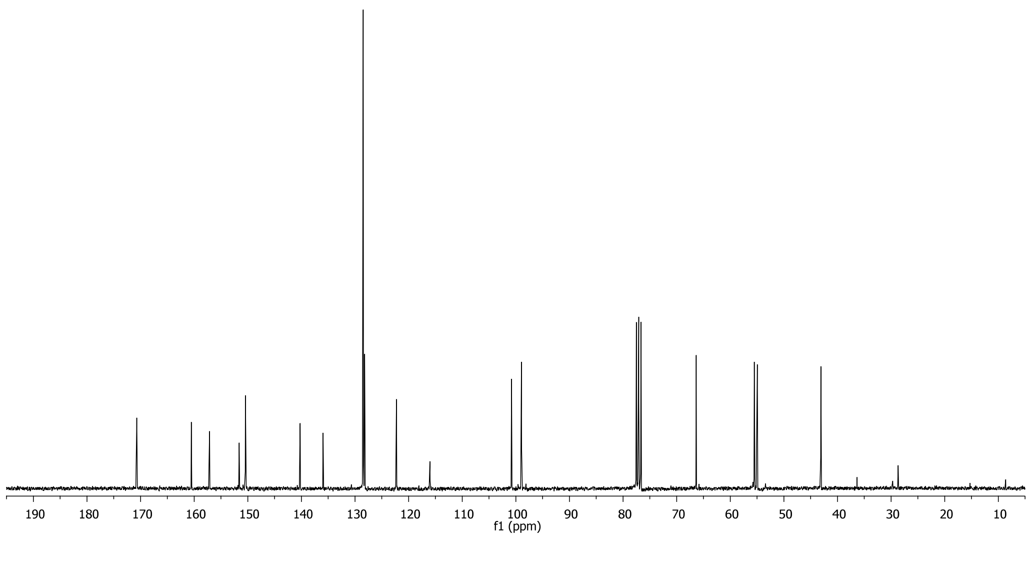
f1 (ppm)

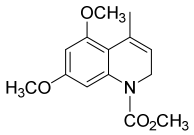
OCH3
H3CO
N
CO2CH3

3a

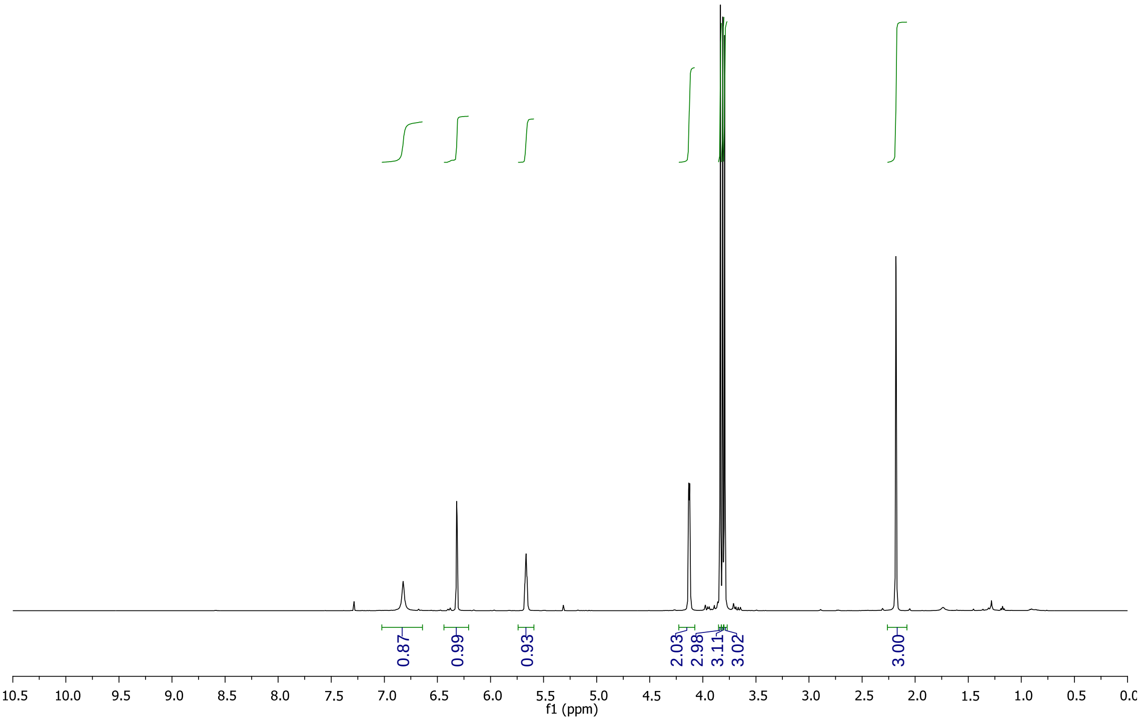
0.87
0.99
0.93
2.03
2.98
3.11
3.02
3.00
10.5 10.0 9.5 9.0 8.5 8.0 7.5 7.0 6.5 6.0 5.5 5.0 4.5 4.0 3.5 3.0 2.5 2.0 1.5 1.0 0.5
f1 (ppm)

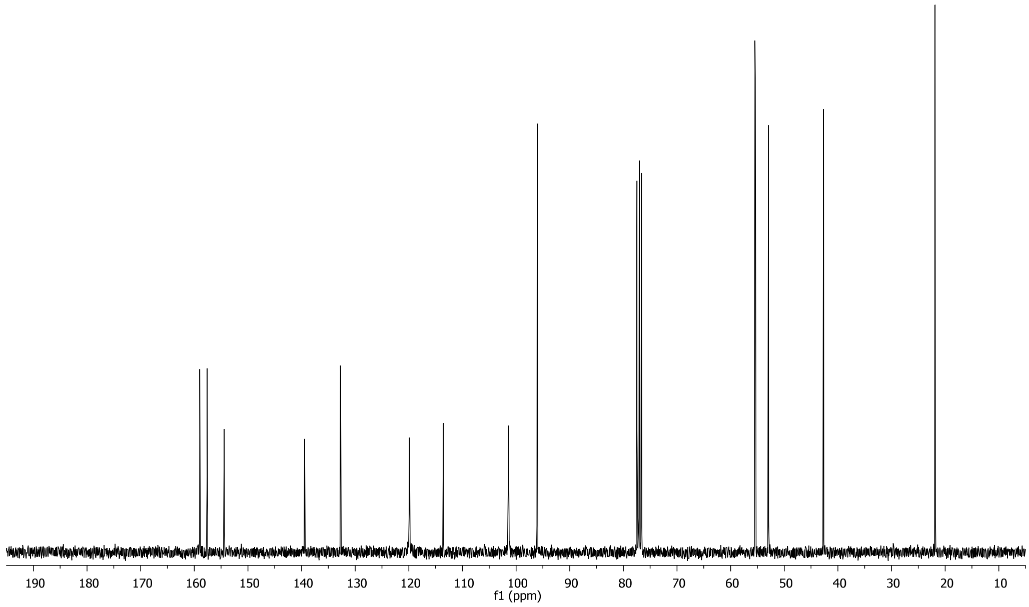
190 180 170 160 150 140 130 120 110 100 90 80 70 60 50 40 30 20 10
f1 (ppm)

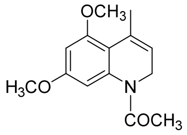
OCH$_3$
H$_3$CO
N
COCH$_3$

3b

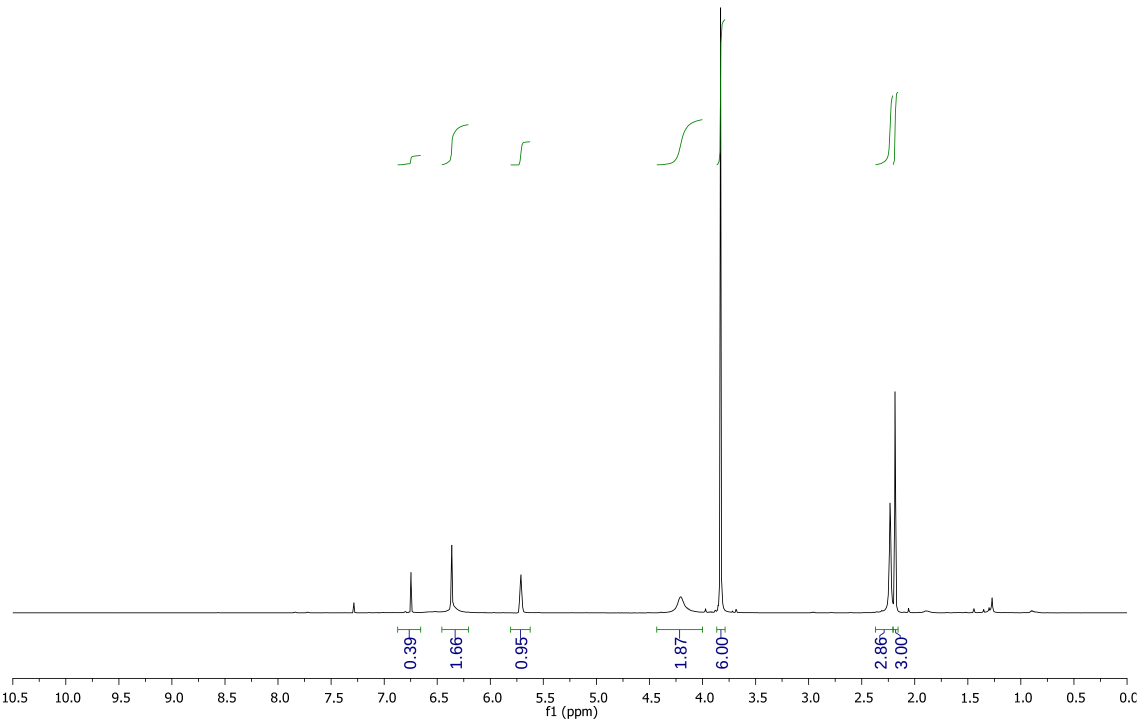
0.39
1.66
0.95
1.87
6.00
2.86
3.00
10.5 10.0 9.5 9.0 8.5 8.0 7.5 7.0 6.5 6.0 5.5 5.0 4.5 4.0 3.5 3.0 2.5 2.0 1.5 1.0 0.5 0.0
f1 (ppm)

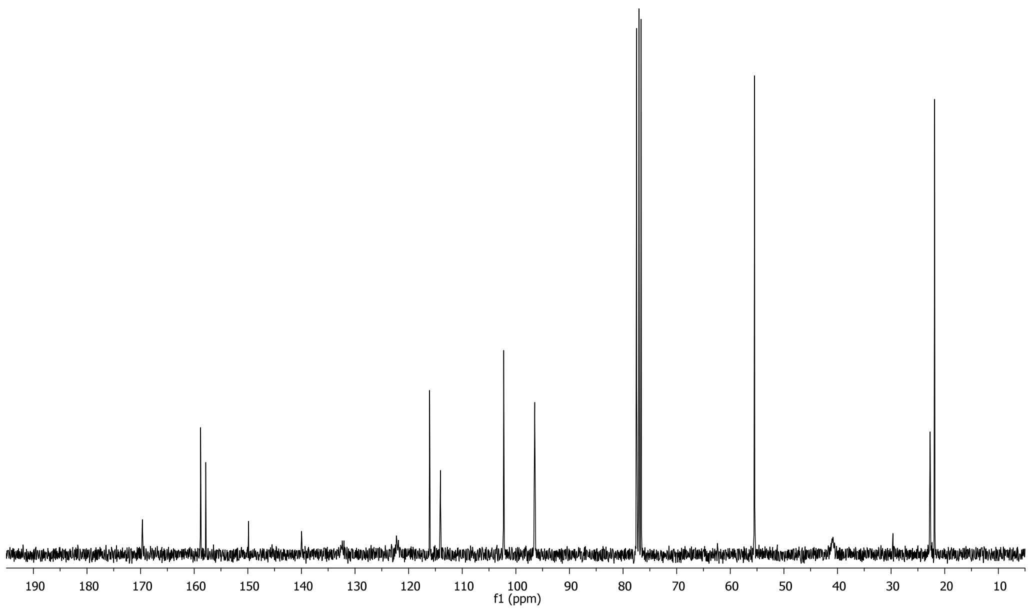
190 180 170 160 150 140 130 120 110 100 90 80 70 60 50 40 30 20 10
f1 (ppm)

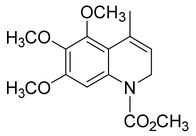
OCH3
H3CO
H3CO
N
CO2CH3

3C

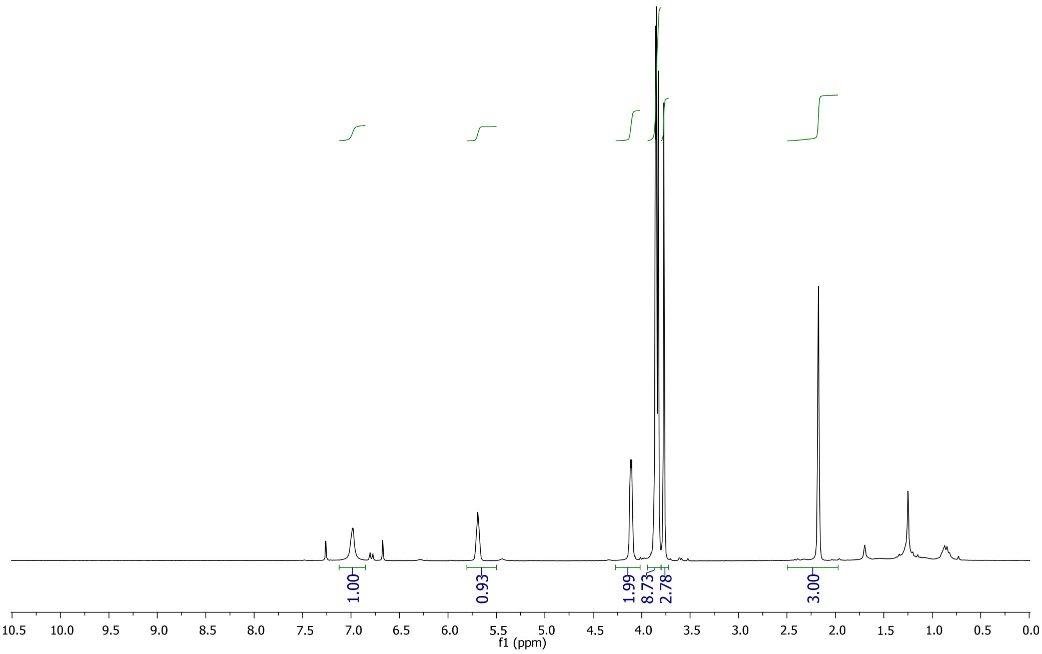
1.00
0.93
1.99
8.73
2.78
3.00
10.5 10.0 9.5 9.0 8.5 8.0 7.5 7.0 6.5 6.0 5.5 5.0 4.5 4.0 3.5 3.0 2.5 2.0 1.5 1.0 0.5 0.0
f1 (ppm)

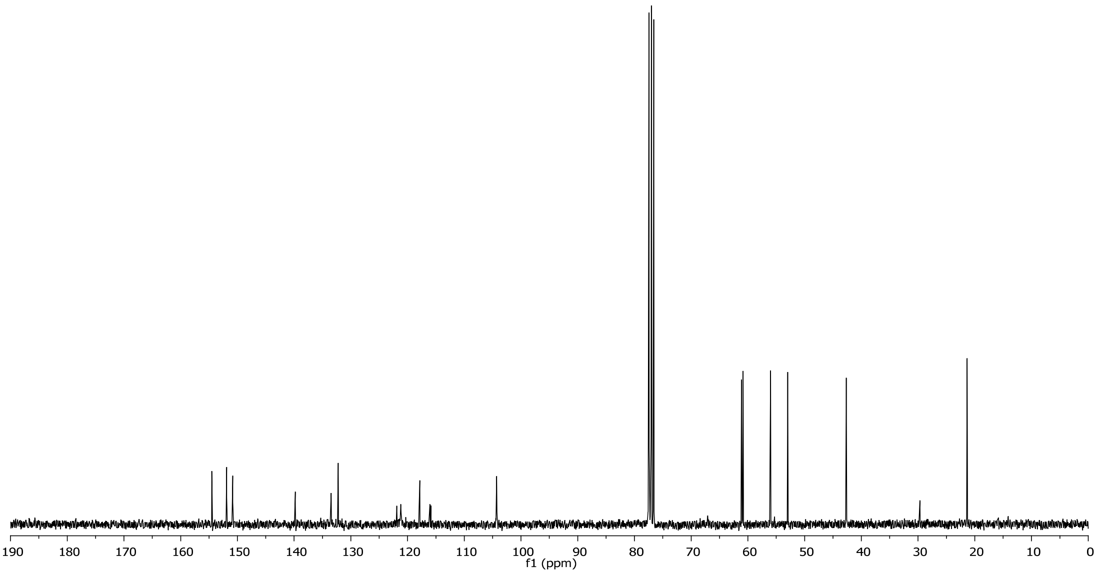
190 180 170 160 150 140 130 120 110 100 90 80 70 60 50 40 30 20 10 0
f1 (ppm)

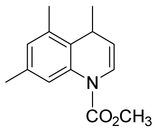

4d

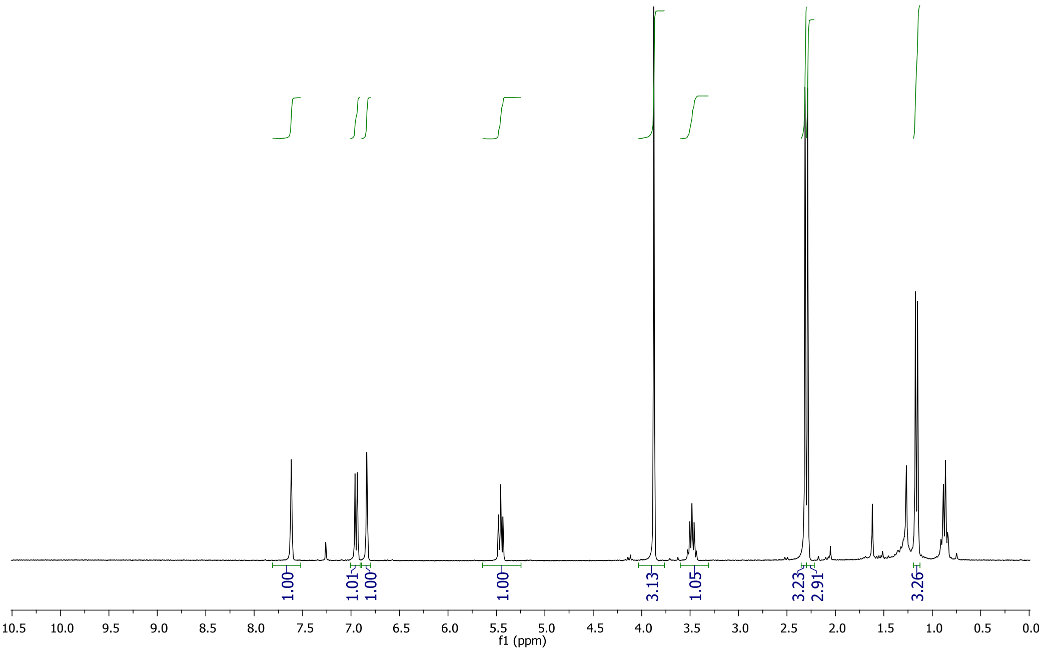

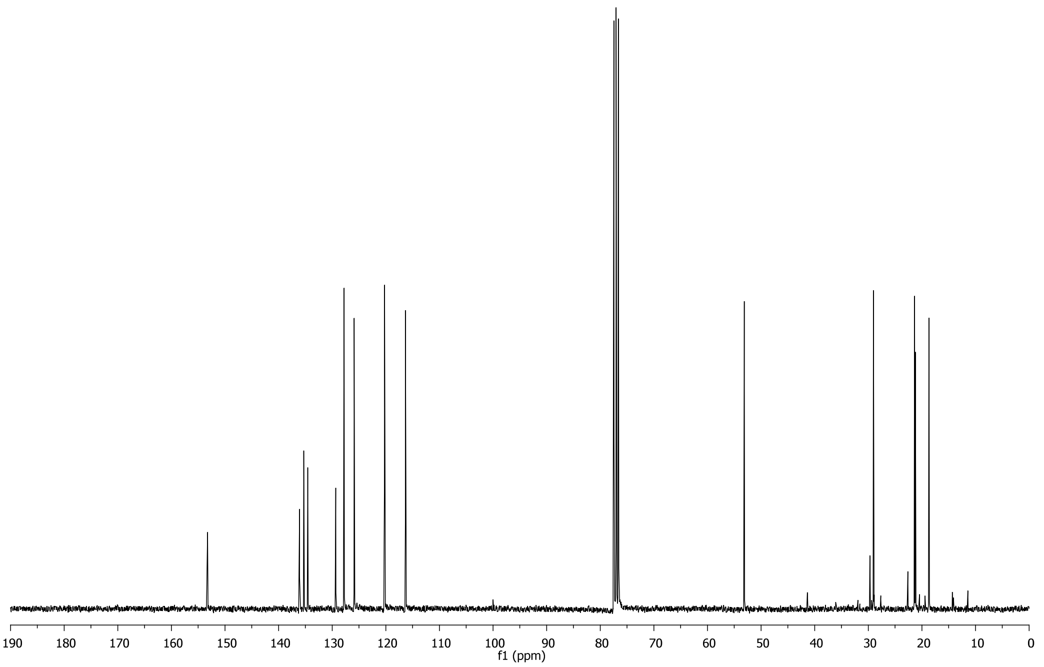

Supplement: Supplementary file 1 [file marinedrugs-15-00276-s001.pdf]
